# Differential Juvenile Hormone Variations in Scale Insect Extreme Sexual Dimorphism

**DOI:** 10.1371/journal.pone.0149459

**Published:** 2016-02-19

**Authors:** Isabelle Mifom Vea, Sayumi Tanaka, Takahiro Shiotsuki, Akiya Jouraku, Toshiharu Tanaka, Chieka Minakuchi

**Affiliations:** 1 Graduate School of Bioagricultural Sciences, Nagoya University, Nagoya, Japan; 2 National Institute of Agrobiological Sciences, Tsukuba, Japan; CNRS, FRANCE

## Abstract

Scale insects have evolved extreme sexual dimorphism, as demonstrated by sedentary juvenile-like females and ephemeral winged males. This dimorphism is established during the post-embryonic development; however, the underlying regulatory mechanisms have not yet been examined. We herein assessed the role of juvenile hormone (JH) on the diverging developmental pathways occurring in the male and female Japanese mealybug *Planococcus kraunhiae* (Kuwana). We provide, for the first time, detailed gene expression profiles related to JH signaling in scale insects. Prior to adult emergence, the transcript levels of *JH acid O-methyltransferase*, encoding a rate-limiting enzyme in JH biosynthesis, were higher in males than in females, suggesting that JH levels are higher in males. Furthermore, male quiescent pupal-like stages were associated with higher transcript levels of the JH receptor gene, *Methoprene-tolerant* and its co-activator *taiman*, as well as the JH early-response genes, *Krüppel homolog 1* and *broad*. The exposure of male juveniles to an ectopic JH mimic prolonged the expression of *Krüppel homolog 1* and *broad*, and delayed adult emergence by producing a supernumeral pupal stage. We propose that male wing development is first induced by up-regulated JH signaling compared to female expression pattern, but a decrease at the end of the prepupal stage is necessary for adult emergence, as evidenced by the JH mimic treatments. Furthermore, wing development seems linked to JH titers as JHM treatments on the pupal stage led to wing deformation. The female pedomorphic appearance was not reflected by the maintenance of high levels of JH. The results in this study suggest that differential variations in JH signaling may be responsible for sex-specific and radically different modes of metamorphosis.

## Introduction

The origin of complete metamorphosis (holometaboly) is a major event in insect evolution, and very likely led to the hyperdiversification of holometabolous insects (flies, butterflies, wasps, and beetles) [1]. These insects develop from a larval stage, undergo complete metamorphosis through a pupal stage, and emerge as a morphologically different imago. Since most juveniles and adult forms use different ecological habitats and food sources, the evolution of complete metamorphosis may have opened new niches for diversification [2, 3]. How has this type of metamorphosis originated? The current phylogenetic framework of insects provides that hemimetabolous paraneopterans (including true bugs), mostly developing through successive nymphal stages, with the progressive development of wings and no change in ecological niche, are the sister group to all Holometabola (flies, butterflies, beetles etc…) and thus, hemimetaboly may have given rise to holometaboly [4]. Therefore, investigating less diverse hemimetabolous groups (such as cockroaches, and grasshoppers) may provide insights into the origin of complete metamorphosis.

A few hemimetabolous insects convergently acquired a unique mode of metamorphosis referred to as neometaboly [4, 5]. The juveniles (nymphs) of these insects first develop through successive molts similar to traditional hemimetaboly, but later enter non-feeding quiescent stages before the imago. The development of wings and adult features occurs during these stages. Neometaboly has been reported in thrips, whiteflies, and male scale insects, which are related groups to holometabolous insects [6]. Therefore, a clearer understanding of how the onset of these quiescent stages is regulated may shed light on the origin of complete metamorphosis.

Insect metamorphosis is governed by two main growth hormones that act in concert: i) juvenile hormone (JH), a sesquiterpenoid produced by the corpora allata (CA) that inhibits metamorphosis by maintaining the juvenile state [7, 8], and ii) ecdysone, a steroid hormone produced by the prothoracic gland that induces each molt [9, 10]. Despite the concomitant discovery of these two hormones, research on their molecular actions have mainly focused on holometabolous insects, such as *Drosophila melanogaster*, *Manduca sexta* and *Bombyx mori*, and mainly on ecdysone (reviewed in Jindra et al. [11]).

Among the reasons for the lack of molecular studies on JH, Methoprene-tolerant (Met), a protein belonging to the bHLH (basic-helix-loop-helix)/PAS (Per-Arnt-Sim) family, was only recently confirmed to be the JH receptor due to its high affinity for binding to JH III in *D. melanogaster* [12] and in *Tribolium castaneum* [13]. The function of *Met* as the JH receptor was also shown in *T. castaneum* by RNAi [14, 15]. Taiman (Tai), another bHLH/PAS protein, was simultaneously identified as a Met co-activator by heterodimerization, and is required for JH signal transduction [16, 17].

The recent development of functional techniques for non-model organisms (RNAi) prompted a number of studies on the molecular role of JH in hemimetaboly (e.g., *Pyrrhocoris apterus* [18], and *Blattella germanica* [19–22]). The findings of these studies have provided unprecedented insights into how genes involved in the hemimetabolous JH signaling pathway orchestrate its function, such as *Krüppel homolog 1* (*Kr-h1*), a JH-inducible and early-response transcription factor in JH signaling [23, 24] as well as *broad* (*br*). For example, *br* is involved in the commitment to the pupal stage in holometabolous insects by its induction from ecdysone concomitant with low JH titer [25]. However, in hemimetabolous insects such as the milkweed bug *Oncopeltus fasciatus* and the cockroach *B. germanica*, *br* responds to JH and promotes wing development across successive nymphal stages [19, 26]. During the post-embryonic development of thrips (Thysanoptera), developing through neometaboly, *br* increases only once, at the onset of the quiescent stage, during which only the wings start to develop [27].

**Fig 1:**
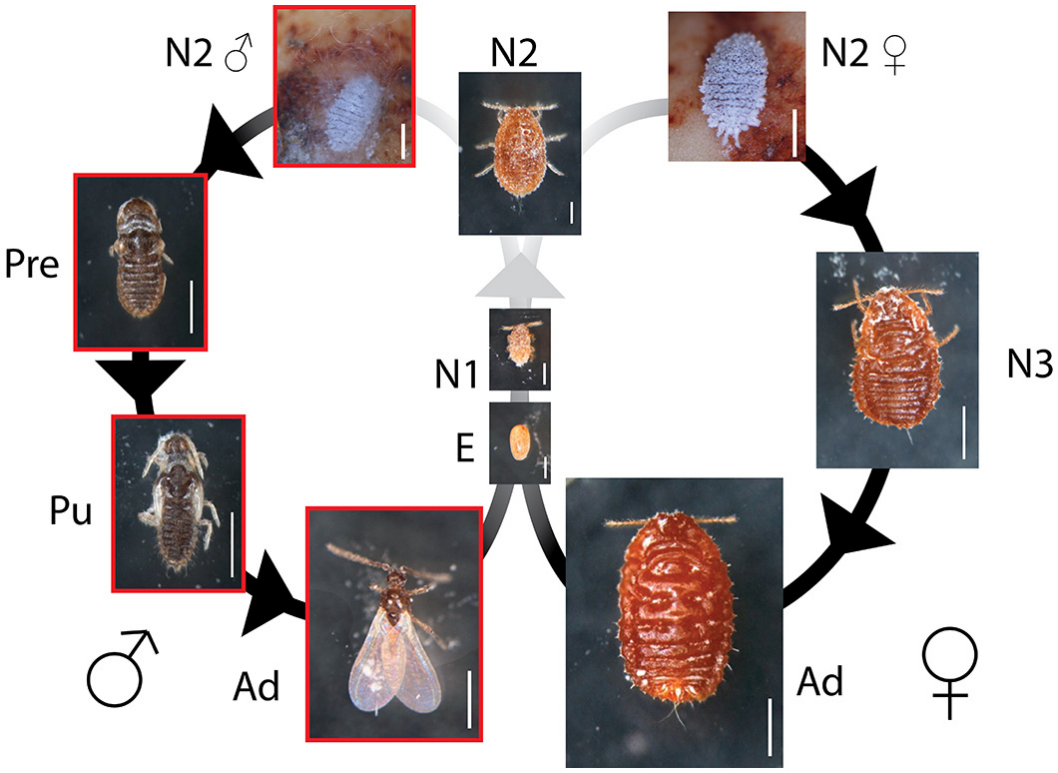
Life cycle of *Planococcus krauhniae*. E: Egg, N1: first-instar nymph (undifferentiated in males and females), N2: second-instar nymph (the first few days are undifferentiated), N2♂: male second-instar nymph (identifiable by filamentous secretions), N2♀: female second-instar nymph (filamentous secretions absent), N3: female third-instar nymph, Pre: male prepupa, Pu: male pupa, Ad: adult. Scale bars: 200 μm (E, N1 and N2), 500 μm (N2♀, N2♂, Pre, Pu, Ad). N1, N2, N3, Pre, Pu, and female adult specimens had their secretions removed in the photographs. Arrows: molting events. Red outline: non-feeding stages. Grey gradient: degree of sexual dimorphism.

Scale insects and mealybugs (Hemiptera: Coccoidea) are important plant pests and are consistently characterized by an extreme sexual dimorphism. The wingless females grow through successive molts and retain juvenile features at the adult stage [28]. In contrast, males not only develop wings but, after a few nymphal molts, they enter two non-feeding quiescent stages (“prepupa” and “pupa”), in which the adult features are formed. Winged adult males show such prominent morphological differences with the females that both sexes do not appear to belong to the same species (Fig 1). This sexual dimorphism is established by divergent post-embryonic developmental pathways after the first-instar nymph. Therefore, growth hormones may be involved in the establishment of these differential pathways. For example, an anatomical study of the soft scale insect *Lecanium corni* (Coccidae) showed that the female CA appears to be significantly larger than the male CA [29]. This finding led Matsuda [30] to conclude that “more JH is secreted (presumably an excessive secretion) in the female than in the male, resulting in the suppression of imaginal structures and concomitant maturation of oocytes because of its gonadotropic function”.

Scale insects present a unique opportunity to examine the JH signaling pathway because they provide two modes of metamorphoses in one study system. On one hand, examining the development of juvenile-like females may contribute to a better understanding of how the JH signaling pathway is involved in permanent aptery and neoteny. On the other hand, investigating the male quiescent stages will provide insights into how JH regulates this rare type of metamorphosis, thereby improving our understanding on the hormonal origin of complete metamorphosis. Finally, the study of two diverging types of metamorphoses in the same study system allows us to directly compare how JH modulations within the same species and same genome influence metamorphosis so radically.

In the present study, we used the Japanese mealybug, *Planococcus kraunhiae* (Kuwana) (Pseudococcidae) and determined whether any differential expression of genes related to JH signaling triggers diverging post-embryonic development between males and females. More specifically, we test whether higher levels of JH in female development compared to the males (as hypothesized by Matsuda), thereby inducing greater levels of *Kr-h1* transcripts, lead them to retain juvenile traits, by preventing metamorphosis.

## Materials and Methods

### Culture conditions

Our Japanese mealybug culture was founded from eggs graciously provided by Jun Tabata (National Institute for Agro-Environmental Sciences, Japan). The insects were subsequently reared at 23°C (16L8D) and fed on sprouted broad beans (Kokusai Pet Food, Kobe, Japan), placed in hermetic containers (12 x 9 cm) with a small opening blocked by a mesh. In order to control humidity levels, filter paper was added to the bottom of the container. Broad beans were replaced every week. Under these conditions, from egg oviposition to adult emergence, development times were approximately as follows: 10 days for the embryonic stage (E) after oviposition, 14-15 days for the first-instar nymphs (N1), 3-4 days for the phenotypically undifferentiated second-instar nymphs (N2), 4 days for the differentiated second-instar nymphs (N2♂ and N2♀), 7-8 days for the female third-instar nymphs (N3), 3 days for the male prepupae (Pre), and 5 days for the male pupae (Pu) (Fig 1).

### Sex ratio estimation and collecting strategy

In mealybugs, during E, N1 and the first 3 days of N2, it is generally not possible to distinguish males and females *in vivo*. However, mealybug sex determination is based on paternal genome heterochromatization in future males [31–33], consisting of the condensation of the paternal genome at the beginning of the embryonic stage [34]. Observable with microscopy, this condensation therefore allows the sex of each embryo to be identified, although they have to be fixed and stained with 4′, 6-diamidino-2-phenylindole (DAPI) beforehand (S1A Fig). This step prevents from identifying the sex and extracting RNA on the same individual. By using DAPI staining in *Planococcus citri*, a previous study found that the overall sex ratio of eggs that a female laid was 1:1 in average, but this ratio varied consistently during oviposition time [35]. In order to determine whether any sex ratio variation occur in *P. kraunhiae*, we collected the eggs of five ovipositing females every 24 hours. Embryos were stained with DAPI as described in Ross et al. [35], with the following modifications: DAPI (Boehringer Mannheim) diluted at 5 μg/mL in PBS was used in our study. Each egg was observed using a Fluorescent Compound Microscope (Olympus BX41) at x200 magnification and sexed, and the average ratio was then calculated for each day.

In order to obtain staged nymphs, fertilized adult females were separated in glass dishes including a broad bean and the eggs were collected every 24 hours and monitored for development in separate glass dishes until they attained the desired stage. They were then collected and pooled in TRIzol reagent (Thermo Fisher Scientific, Inc.) for RNA extraction. Samples from E to N2D3 were collected using the sex-bias strategy resulting from sex ratio variations observed during oviposition.

### Identification of sequences and cDNA cloning

The total RNA of pooled individuals from different stages was extracted using TRIzol reagent following the manufacturer’s manual with glycogen precipitation. Samples were collected, homogenized with a pestle in 50 to 100 μL TRIzol, then flash frozen at −80°C until processed for RNA extraction. The final amount of RNA could not be assessed because of the presence of glycogen. Oligo-dT-primed reverse transcription was then performed using the PrimeScript II 1^st^ strand cDNA synthesis kit (Takara Bio, Shiga, Japan) using 8 μL of RNA template. In the present study, we examined the expression profiles of genes known to be involved in the hemimetabolous insect JH signaling pathway: *Met*, *tai*, *Kr-h1* and *br*. We also assessed the expression of the *juvenile hormone acid O-methyltransferase* (*jhamt*), encoding for an enzyme involved in the last steps of JH biosynthesis [36–39]. Primers for *br*, *Kr-h1*, *jhamt*, *Met*, *tai* and the *ribosomal protein L32*(*rpL32*, reference gene) were designed after identifying the putative homologous sequences from an unpublished transcriptome database (RNA-Seq) of the Japanese mealybug (see S1 Text) and these fragments were then cloned using the RT-PCR approach (for a detailed protocol, see S1 Text). The conserved region sequences of all investigated genes, were first identified using the transcriptome. Primers were then designed to perform 5′ and 3′ end RACE using a SMARTer RACE cDNA Amplification Kit (Clontech) in order to obtain full-length cDNA sequences (see S1 Text). Primer sequences are listed in S1 Table. All PCR products were purified and cloned into the pGEM-T Easy Vector (Promega, Madison, WI) and sequenced. DNA sequence data were deposited in the DDBJ/EMBL-Bank/GenBank International Nucleotide Sequence Database with the following accession numbers: *Pkjhamt* (LC055463); *PkMet* (LC055464); *Pktai* (LC068770-LC068773); *PkKr-h1* (LC075597 and LC075598); *Pkbr* (LC055465- LC055479); *PkrpL32*(LC055462).

### Quantitative real-time RT-PCR

Total RNA was extracted from all samples using the sex-bias collecting strategy as described above. Each sample consisted of 0.5 to 2 mg of pooled individuals homogenized in TRIzol reagent with glycogen precipitation, and reverse transcribed using the PrimeScript RT reagent Kit with the gDNA Eraser (Takara Bio). Expression profiles for the post-oviposition development of males and females were established by quantifying the levels of transcripts for targeted fragments using absolute quantitative RT-PCR performed on a Thermal Cycler Dice Real Time System (model TP800, Takara Bio) as described previously [27]. Primer sequences are listed in S1 Table. Serial dilutions of a plasmid containing the ORF of each gene were used as standards. The values obtained by the second derivative maximum (SDM) method were normalized with the *rpL32* levels of the transcripts. All the analyses performed using qRT-PCR data are detailed in S1 Text.

### JHM treatments

JHM treatments were performed with pyriproxyfen. Groups of five males staged at PreD1 (24-48 h after molting to the prepupal stage) were placed on a filter paper (Toyo Roshi, Japan), and 5 μL of pyriproxyfen (dissolved in methanol at the concentrations of 1 and 5 mM) was applied on the prepupae. Excess chemical solution was immediately absorbed by the filter paper. After the prepupae started to move again, they were transferred into 1.5-mL tubes, in which filter paper with 10 μL of distilled water was added to maintain humidity. The prepupae were monitored until adult emergence (males stop feeding from the end of N2) and surviving males were transferred to separate glass dishes, including a virgin adult female feeding on a sprouted bean to establish whether they were capable of reproducing. We also observed any phenotypic differences and measured the gene expression between the control and treated samples. To measure the gene expression levels, we proceeded to the same treatment protocol, except that each group of 4-6 PreD1 was pooled for RNA extraction 5 days after the treatment. RNA extraction and cDNA synthesis for quantitative RT-PCR were performed as described above. The same approach was used for treatments on PuD0 (0-24 h after molting to the pupal stage), and RNA was extracted 6 days after the treatment. For each of the treatment, we made five and six biological replicates (for prepupal and pupal treatments respectively), each of them consisting of three to six male pupae pooled for RNA extraction.

## Results

### Collecting strategy for developmental profiles

Based on the DAPI stained embryos, the sex ratio variation in *P. kraunhiae* follows a similar pattern with that in *P. citri* [35]. The highest bias for male embryos occurs during the first two days of oviposition (OD1 and OD2) and the highest bias towards female embryos during the fourth and fifth days of oviposition (OD4 and OD5) (S1B Fig). Therefore, our collecting strategy for samples between E and the middle of N2 consisted of isolating eggs laid during the first two days of oviposition (OD1 and OD2) for male-biased samples, and eggs laid during the fourth and fifth oviposition day (OD4 and OD5) for female-biased samples.

### Expression profiles of JH biosynthetic and signaling genes during male and female development

**Fig 2:**
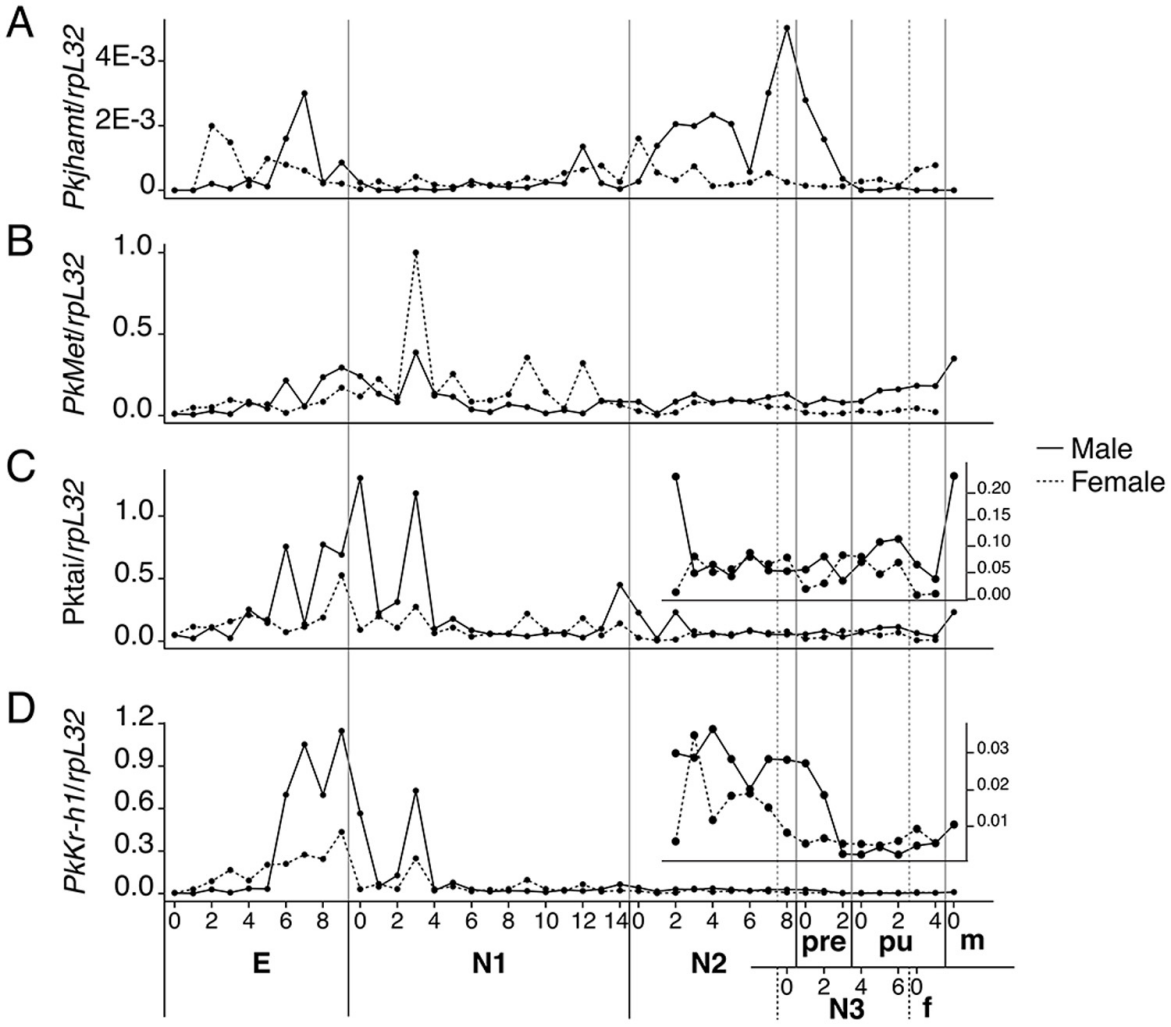
JH biosynthesis, JH signal transduction, and early responses. JH biosynthesis, JH signal transduction and early response. Expression profiles of *Pkjhamt* (A), *PkMet* (B), *Pktai* (C), and *PkKr-h1* (D) during male and female mealybug development after egg oviposition. E: Egg, N1: first-instar nymph, N2: early second-instar nymphs, N3: female third-instar nymph, f: female adult, pre: male prepupa, pu: male pupa, m: male adult. Samples were collected from E to N2D3, using the sex-bias strategy (see the Methods section).

We examined the expression of *Pkjhamt* as an indicator of JH biosynthesis. A phylogenetic reconstruction with the JHAMT protein sequences of other insects confirmed that the *Pkjhamt* that we cloned was homologous to this enzyme (S2 Fig). *Pkjhamt* was initially expressed in male-biased and female-biased samples during the embryonic stage. However, *Pkjhamt* in males only increased approximately 5 days after oviposition, while its expression in females started early after the day of oviposition (Fig 2A). *Pkjhamt* remained at low levels after hatching until the beginning of N2 in male-biased samples. Its expression rapidly increased and peaked towards the end of N2♂ (28 days after oviposition) (Fig 2A), and then decreased before stopping at the beginning of Pu. In contrast, after the embryonic stage in females, *Pkjhamt* remained at relatively low levels throughout the rest of the post-embryonic development.

Met and Tai, by heterodimerization, form the active receptor of JH. PkMet included the bHLH and PAS domains (S3A Fig). The relative levels of *PkMet* transcripts increased in the last days of the embryonic stage, and then were higher in female-biased N1, but remained equivalent in both sexes during N2 until immediately before molting to N3 or Pre. *PkMet* increased slowly from Pre in males until they emerged as adults. In females, *PkMet* expression decreased and stayed at low levels until the adult stage (Fig 2B).

5′ and 3′ end RACE allowed us to identify two isoforms on both the N- and C-termini of *Pktai* (S3B Fig). On the C-terminus, the two isoforms resulted from an indel region (INDEL-1) previously identified in *B. germanica* and *T. castaneum*. The isoforms identified on the N-terminus (S3B Fig; *Pktai* 5A and 5B) differed in the region preceding the bHLH domain. We measured the levels of transcripts for five regions of *Pktai*: the common region (*Pktai* com), 5′ A region (*Pktai* 5A), 5′ B region (*Pktai* 5B) and the indels on the 3′ end (*Pktai* IN-1 and *Pktai* DEL-1). *Pktai* com mRNA levels were the highest at the end of the embryonic stage and beginning of N1, especially in male-biased samples. However, no distinct expression pattern was observed after the middle of N2 between males and females (Fig 2C). The expression of *Pktai* IN-1 was generally higher than *Pktai* DEL-1 by a factor of 10 to 20. However, differences in expression after N2D3 were more evident, with an expression pattern similar to *PkMet* mRNA (increase in males and decrease in females) (S3C Fig). The expression of *Pktai* 5A and *Pktai* 5B differed during the embryonic stage, with that of *Pktai* 5B, starting in late embryos, whereas the expression after N2 was similar to *Pktai* IN-1 (S3D Fig).

We identified two isoforms at the 5′ end of *PkKr-h1* (here mentioned as *PkKr-h1* com for the common region and *PkKr-h1* A and *PkKr-h1* B for each isoform). *PkKr-h1* B consisted of a 294-bp deletion on the 5′ end, and the translated amino acid sequence of *PkKr-h1* B was shorter than that of *PkKr-h1* A by 34 amino acids (S4 Fig). The expression of *PkKr-h1* com (Fig 2D) and *PkKr-h1* A followed a similar pattern (S5A Fig), with (i) high transcript levels during the embryonic stage and the beginning of N1, and (ii) diverging expression between males and females starting during N2. *PkKr-h1* was maintained throughout N2♂, dropped rapidly during Pre, then remained at low levels in Pu. In contrast *PkKr-h1* continuously decreased throughout N2♀, and was maintained at low levels during N3. The transcript levels of *PkKr-h1* B were significantly lower in males and females (S5B Fig), suggesting that it may be a minor isoform.

### *PkKr-h1 A* is involved in JH signaling

**Fig 3:**
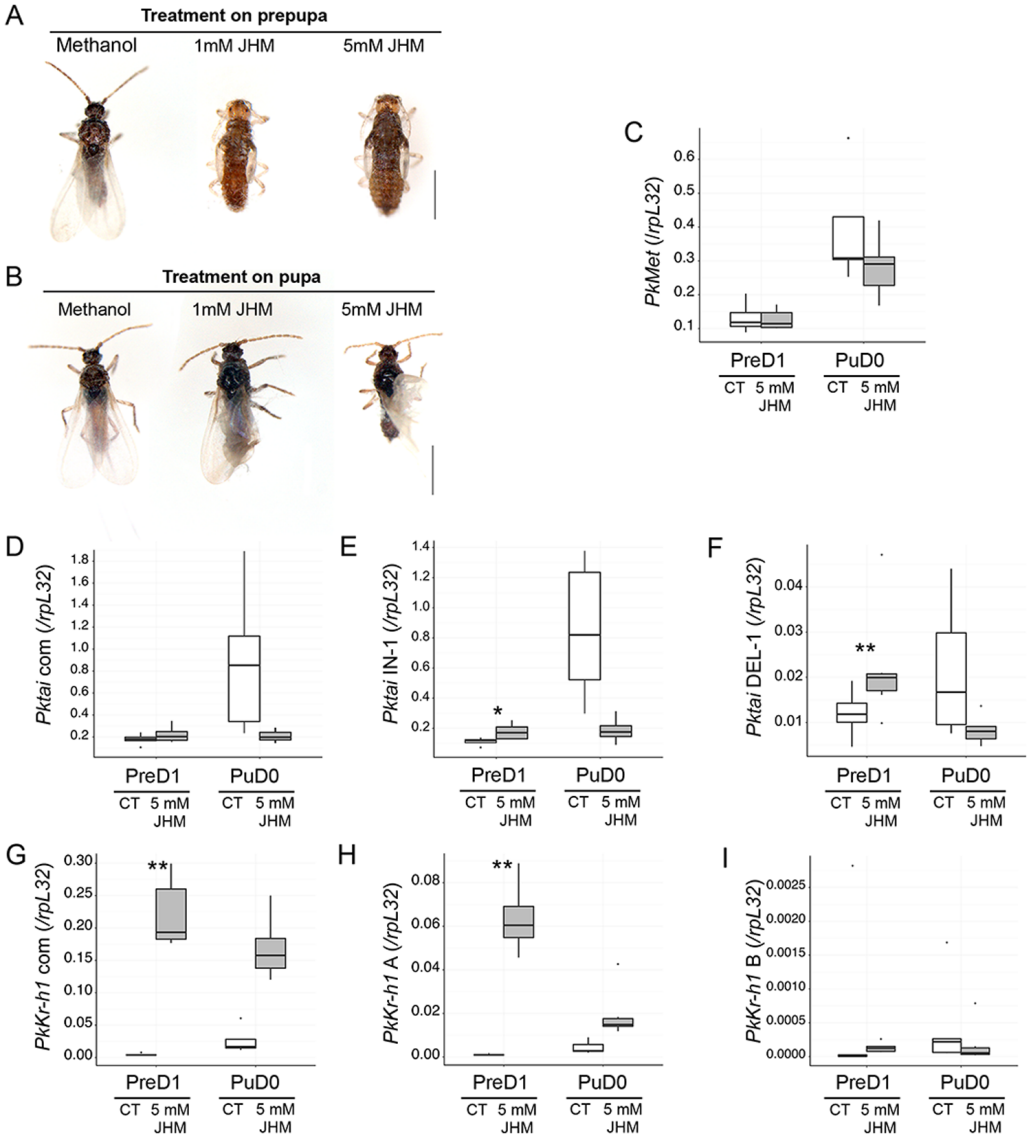
JHM pyriproxyfen effects on male development and JH signaling. A. Treatment during prepupa. B. Treatment during pupa. (C-I) Effects of JHM on gene expression. PreD1: Treatment 24-48 hours after the prepupal molt, RNA extraction 5 days after the treatment, N = 6. PuD0: Treatment 0-24 hours after the pupal molt, RNA extraction 6 days after the treatment, N = 5. Boxplots were constructed with the ggplot2 R package (with upper and lower hinges: 1st and 3rd quartiles, middle line: median, dots: outlier values). Unpaired two-sample Student’s t-test with *: p-value < 0.05 and **: p-value < 0.01. C. *PkMet*; D. *Pktai* com; E. *Pktai* IN-1; F. *Pktai* DEL-1; G. *PkKr-h1*; H. *PkKr-h1* A; I. *PkKr-h1* B.

The treatment with the JHM (pyriproxyfen) during the prepupal stage resulted in a delay in adult molting. JHM-treated male prepupae molted to normal pupae after 3 days. However the following molt resulted in a second pupal stage (Pupa II) after 2 or 3 days and the latter never emerged as adults (Fig 3A, Table 1). Pupae II that were then kept with virgin females did not result in oviposition as opposed to the control treatment. JHM-treated male pupae resulted in variations of adults with different degrees of wing developmental defaults, whereas other features were developed normally (Fig 3B, Table 1). In this case, JH- and methanol-treated males that were subsequently kept with virgin females resulted in oviposition.

**Table 1:** Effects of the pyriproxyfen (JHM) treatment on male metamorphosis in *P. kraunhiae*.

Hormonal treatment		N	Number of dead individuals (%)		Pupa II	Deformed adult	Normal adult
Treated stage	Compound		Prepupa	Pupa			
Prepupa	Methanol	40	9 (22.5)	1 (2.5)	0 (0)	0 (0)	30 (75)
	1 mM JHM	27	4 (15)	0 (0)	23 (85)	0 (0)	0 (0)
	5 mM JHM	31	7 (22.6)	2 (6.5)	22 (70.9)	0 (0)	0 (0)
Pupa	Methanol	38	NA	9 (23.7)	0 (0)	0 (0)	29 (76.3)
	1 mM JHM	23	NA	2 (8.7)	0 (0)	19 (82.6)	2 (8.7)
	5 mM JHM	39	NA	4 (10.3)	0 (0)	29 (75.4)	5 (15.3)

NA: Non-applicable.

JHM did not significantly change the expression of *PkMet* (Fig 3C) or *Pktai* com (Fig 3D), even though *Pktai* IN-1 (p-value<0.05; S2 Table) and *Pktai* DEL-1 (p-value<0.01; S2 Table) significantly increased in JHM-treated PreD1. A decrease in average expression was observed for JHM-treated PuD0 but not significant (Fig 3E and 3F). *PkKr-h1* com and *PkKr-h1* A expression increased significantly (both p<0.01; S2 Table) in treatments performed on PreD1 only (Fig 3G and 3H). In PuD0 treatments, *PkKr-h1* increased but not as markedly as for the prepupal stage, and not considered to be significant.

### *Pkbr3* is involved in JH signaling

**Fig 4:**
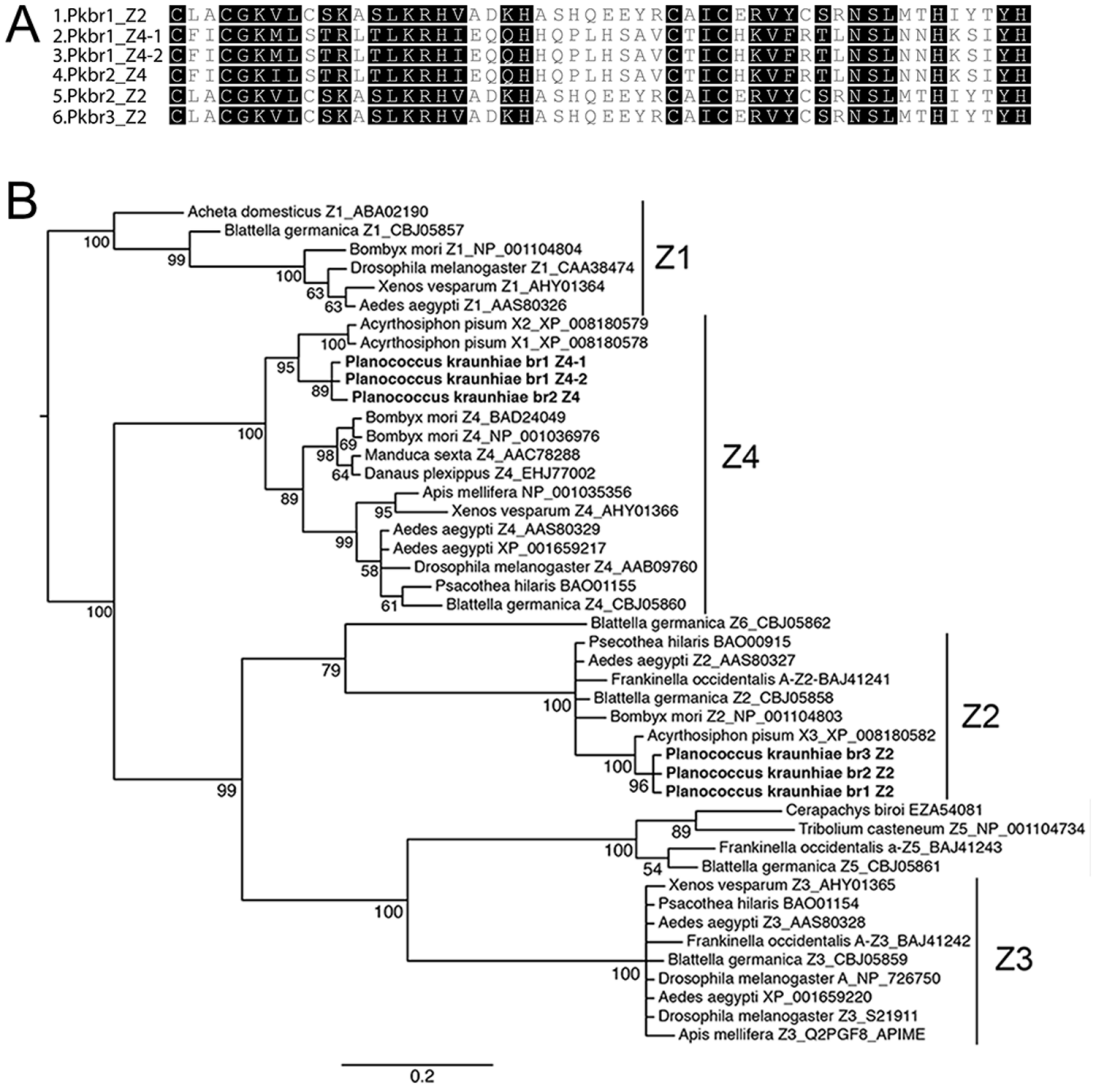
*br* copies and zinc-finger isoforms in the Japanese mealybug. A. Protein alignment of the zinc-finger motif for each identified isoform. B. Phylogeny of zinc-finger isoforms for different insect groups. See Supporting Information for the phylogenetic analysis method.

While examining the transcriptome of the Japanese mealybug, three highly similar copies of *br* were identified (S6A Fig) and described as *Pkbr1*, *Pkbr2* and *Pkbr3* hereafter. 3′ RACE identified different isoforms with and without a zinc-finger motif for each of the three copies (S6B–S6D Fig). A phylogenetic tree constructed based on the protein sequence alignment of each zinc-finger isoform (Fig 4A) identified them as homologous to Z2 (*Pkbr1* Z2, *Pkbr2* Z2 and *Pkbr3* Z2), and Z4 (*Pkbr1* Z4-1, *Pkbr1* Z4-2 and *Pkbr2* Z4) (Fig 4B). Although *P. kraunhiae* genome is not available at this moment, the *Pkbr* copies we identified are likely to be recent paralogs and each of them encode for at least Z2 and Z4 isoforms.

**Fig 5:**
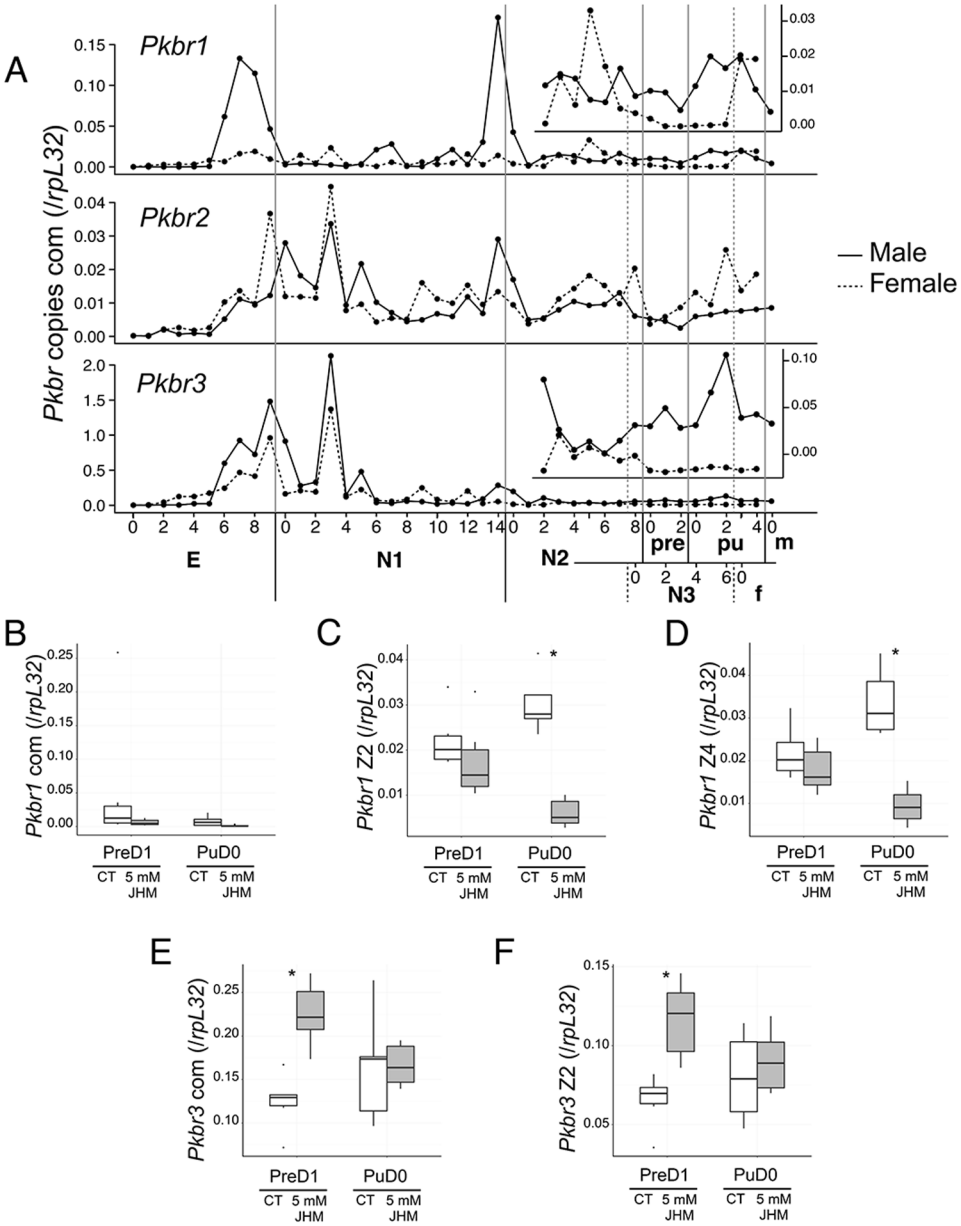
Expression profiles and JHM effects on *br*. A. Expression profiles of *Pkbr* copies identified in the present study. E: Egg, N1: first-instar nymph, N2: second-instar nymphs, N3: female third-instar nymph, Pre: male prepupa, Pu: male pupa. Samples were collected using the sex-bias strategy from E to N2D3. B-F. Effects of JHM on gene expression. PreD1: Treatment 24-48 hours after the prepupal molt, RNA extraction 5 days after the treatment, N = 6. PuD0: treatment 0-24 hours after the pupal molt, RNA extraction 6 days after the treatment, N = 5. Boxplots were constructed with the ggplot2 R package (with upper and lower hinges: 1st and 3rd quartiles, middle line: median, dots: outlier values). Unpaired two-sample Student’s t-test with *: p-value < 0.05 and **: p-value < 0.01. B. *Pkbr1* com, C. *Pkbr1* Z2, D. *Pkbr1* Z4, E. *Pkbr3* com, F. *Pkbr3* Z2.

We examined the expression profiles of each of the zinc-finger isoforms, along with the common region of each copy. In males, the expression of the *Pkbr1* common region (*Pkbr1* com) peaked before hatching and at the molting event from N1 to N2, then remained at equivalent levels after N2. In the case of females, the levels of *Pkbr1* mRNA were consistently low (Fig 5A, top). *Pkbr1* Z2 and Z4 showed a similar pattern to *Pkbr1* (S7A and S7B Fig). *Pkbr2* com and its isoforms were expressed at significantly lower levels than the other copies and did not show any sex-specific patterns (Fig 5A middle, S7C and S7D Fig). The expression profile of *Pkbr3* mRNA presented the most similar pattern to that of *PkKr-h1*, with the highest levels being observed during the embryonic stage and beginning of N1, and a divergence in expression between males and females at the very end of N2. However, *Pkbr3* expression levels continued to increase and peaked during the pupal stage (Fig 5A bottom). This pattern was similar to that of *Pkbr3* Z2 (S7E Fig).

A JHM treatment on PreD1 did not affect the expression of *Pkbr1* or its isoforms (Fig 5B), while this treatment performed on PuD0 induced a significant decrease in *Pkbr1* Z2 and *Pkbr1* Z4 (Fig 5C and 5D; p < 0.01 and 0.05 respectively, S2 Table). *Pkbr3* and *Pkbr3* Z2 expression significantly increased with the prepupal treatments (p < 0.01, S2 Table), whereas the increase observed for the pupal treatment was not significant (Fig 5E and 5F). *Pkbr2* and its isoforms were not affected by the JHM treatment (S8 Fig). Taken together, these results suggest that *Pkbr3* is the copy involved in the JH signaling pathway, and may be induced by the expression of *PkKr-h1*.

## Discussion

Scale insects exhibit two types of sex-specific metamorphoses, the investigation of which has implications in the understanding of: 1) the origin of complete metamorphosis with male quiescent stages, and 2) the underlying mechanisms of pedogenesis in females. In the present study, we followed the development of each sex and assessed the expression of genes known to be involved in the insect JH signaling pathway.

We provide, for the first time, the *Pkjhamt* expression profile in scale insects, a gene that codes the enzyme acting in the last step of JH biosynthesis [36, 39]. We favored indirect measurements of JH titers because, given the minute size of the early developmental stages, a daily direct measurement was challenging. PkJHAMT enzymatic activity still needs to be assessed. However, based on our expression results, *Pkjhamt* transcript levels are consistent with *PkKr-h1*. Moreover, a phylogenetic analysis of the protein confirmed PkJHAMT homology to other functional JHAMT in insects (S2 Fig).

We herein also present a detailed account of *Met* and *tai* expression. Met and Tai have recently been confirmed as the functional receptor complex of JH [11]. The post-embryonic expression of *Met* and/or *tai* has been previously assessed in hemimetabolous cockroaches (*B. germanica*: *Met* [21]; *B. germanica*: *tai* [20]), true bugs (*P. apterus*: *Met* [18]), but also in wax scale insects (*Ericerus pela*: *Met* and *tai* [40]) at different developmental stages. Our expression profile differs from wax scale insect development, with *Met* expression increasing during N2♂ and being maintained at higher levels than those in females through the end of development. However, direct comparisons may not be possible because only stages were sampled in Yang et al. [40] while our study used a 24-hour sampling strategy. Although *tai* expression did not appear to differ between male and female development, a careful study of putative *tai* isoforms [20] may highlight any sex-specific differential expression.

*Kr-h1*, a transcription factor containing several C2H2-type zinc-fingers [41], is induced downstream of *Met* as an early-response gene of JH [24] and has been shown to be functionally essential for the embryonic development of *Drosophila* [42]. In *Drosophila*, three isoforms have been identified at the N-terminus and Kr-h1 a is responsible for metamorphosis [24, 43]. Two isoforms at the 5′ end have been identified in *B. mori* [44]. In thrips, three isoforms at the 5′ end were also identified, although they did not differ in the ORFs [27]. In the present study, we identified two isoforms, *PkKr-h1* A and *PkKr-h1* B, which differed by the deletion of 34 amino acids in *PkKr-h1* B. However, the general expression pattern suggested that *PkKr-h1* B is a minor isoform.

We also identified three presumably paralogous copies of *Pkbr*, with *Pkbr1* and *Pkbr2* having at least isoforms, Z2 and Z4, with *Pkbr3* possessing at least the Z2 isoform. Only one copy of *br* has been identified in other insects so far to date. Given the high similarity between the isoforms of each copy, we suggest that *Pkbr* 1, 2, and 3 are paralogs and that only *Pkbr3* plays a role in JH signaling. In *B. germanica*, Z2 is the most abundant isoform [19], and this may explain why we easily identified this isoform for the *br* gene three copies.

**Fig 6:**
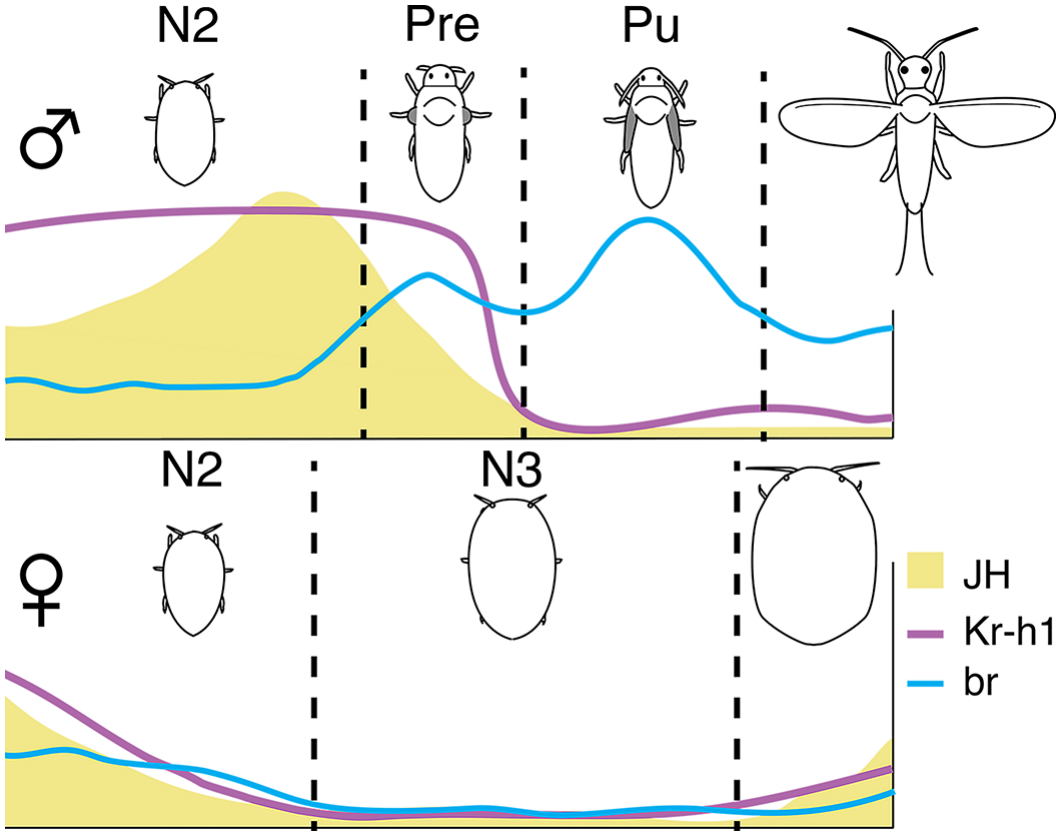
Summary of differential JH titers and early response gene patterns when phenotypic differences arise during post-embryonic development in *P. kraunhiae*. N2: Second-instar nymph, N3: female third-instar nymph, Pre: prepupa, Pu: pupa.

We generally found that JH-induced genes were expressed during 1) the embryonic stage and 2) towards the middle of N2 in males. The expression patterns of *PkKr-h1* and *Pkbr3* during the embryonic stage corroborates with previous findings on thrips (*Kr-h1* and *br* [27]), true bugs (*Kr-h1* and *br* [18]), and *B. germanica* (*br* only [45]), in which the expression of *Kr-h1* and *br* are the highest during the second half of the embryonic stage, and *Pkbr3* continues to be expressed at the onset of N1. Thereafter, although not as high as during the embryonic stage, the expression of *PkKr-h1* starts to differ by its decrease in N2♀, while it increases in N2♂, and drops at the end of prepupa. This general expression pattern at least in males, is similar to that of thrips, an insect group basal to holometabolous insects and with a similar mode of metamorphosis to male scale insects [27]. Based on *Pkjhamt* and downstream gene expression levels, we suggest that sexual dimorphism in scale insects is established by a divergence in JH titers, at the middle of N2, resulting in opposite expression levels of the first response genes in JH signaling (Fig 6), which triggers different pathways in development towards the adult stage in males and females.

### Male quiescent stages and wing development

The function of Br in wing development, more specifically wing size and shape, has been demonstrated in a few instances in hemimetabolous insects [19, 26] and also in the basal holometabolous red flour beetle [15, 46, 47], and this function also appears to be present in highly derived holometabolous insects such as *Drosophila*. Previous studies reported that a *Drosophila* mutant lacking *br* arrested at the end of the larval stage [48, 49], suggesting that *br* is a key specifier of pupal development [50].

In the present study, the expression pattern of *Pkbr3* was more similar to that of *PkKr-h1* than to the other *br* copies, with *Pkbr3* in males continuing to increase even after *PkKr-h1* levels decreased at the end of the prepupal stage. Applying JHM at the onset of the prepupal stage prolonged the expression of *PkKr-h1* and *Pkbr3* to the pupal stage and disrupting adult metamorphosis, thereby reinforcing that JH plays a role in male quiescent stages through *PkKr-h1* and *Pkbr3* in scale insects. However, functional studies using RNAi techniques will provide a better understanding of the role of each *Pkbr* copy and isoform.

The *Pkbr3* expression pattern is more similar to that of the last instar and adult stages of *P. apterus* than the *br* pattern of thrips, with levels decreasing in the same manner as *Kr-h1* in the latter [18]. The phylogenetic affinity between true bugs (Heteroptera) and scale insects, both of which belonging to Hemiptera, may explain this similarity. More importantly, our results can imply and we hypothesize that thrip and male scale insect neometaboly, although phenotypically similar, do not follow the same regulatory mechanisms, thereby corroborating with a convergent origin of this type of metamorphosis.

We found that *Pkbr1* expression peaked at the end of the embryonic stage and N1 in male-biased samples. Despite the high similarity of BTB protein sequences, the RNA transcripts levels differ from each other and *Pkbr1* isoforms responded significantly and negatively to JHM only if treated during the pupal stage, suggesting a role in development that differs from the JH pathway. Further studies on the transcriptional regulation of *Pkbr* by ecdysone will provide deeper insights into the role of *Pkbr1*.

### Female neoteny

The maintenance of juvenile features in adults, through neoteny, has evolved independently at least 6 times in insects [51]. Sexual dimorphism with sex-specific neoteny has also been reported in female Strepsiptera and a study on the expression of *br* isoforms suggested that the suppression of pupal determination (by the absence of the induction of *br* com and *br* Z3) contributed to female neoteny [52]. In scale insects, female pedogenetic development, characterized by the lack of wings and other adult features found in males, is a key characteristic of this diverse group [28] with no later reversion, suggesting that this form of development in female scale insects is advantageous for plant parasitism and also that the mechanism underlying the retention of juvenile traits may be highly conserved. Although it is plausible to consider that more than one mechanism for the establishment of neotenic adult has arisen in scale insect evolution, fossil records [53] and current diversity [28] provide solid evidence that scale insect juvenile-like adult females only appeared once. *Kr-h1* levels in a wax scale insect were assessed at each developmental stage except for the embryos and highest levels were measured in FA3 (late female adult) when oocytes had already developed and were possibly already fertilized. *PkKr-h1* was the lowest during FS (female second-instar) [40].

Despite previous assumptions that female neoteny is caused by higher titers of JH produced by larger CA in scale insects, based on the study of *Lecanium corni* (Coccidae) [30, 54], our results show an opposite pattern with a decrease in *Pkjhamt* and *PkKr-h1* at the onset of the female last juvenile instar (N3), with low levels being maintained up to the adult molt. The low levels of *PkKr-h1* and *Pkbr3* during the end of female development however are consistent with the lack of wing development. Recent studies have suggested a link between *Kr-h1* and *E93*, a transcription factor directly linked to adult specification [55, 56]. Preliminary assessments are showing that *E93* is involved in female neoteny (Vea et al., in prep.) and more detailed functional studies on the transcription factor and related genes will provide a clearer understanding of how low levels of *PkKr-h1* contribute to the pedomorphic development of female scale insects.

## Supporting Information

S1 Text Protocols for RT-PCR and RACE and R analyses. (PDF)

S1 Fig Sex ratio variations in *Planococcus kraunhiae* during oviposition. A. Photograph of fixed and DAPI-stained embryos at oviposition, top: male embryo as evidenced by the bright, condensed paternal genome, bottom: female embryo as evidenced by homogeneous nuclei. B. Percentage of male embryos on each oviposition day. Counts were made using DAPI-stained embryos collected every 24 hours from fertilized females (N = 5) and observed an Olympus BX41 Fluorescence microscope (x200).
(JPG)

S2 Fig Phylogeny of JHAMT protein sequences obtained with MrBayes 3.2.5. GenBank accession numbers: *Acyrthosiphon pisum*: NP_001156251, *Aedes aegypti*: EAT42177, *Apis mellifera*: AGG79412, *Bombyx mori*: NP_001036901, *Drosophila melanogaster*: AAF53533, *Nasonia vitripennis*: XP_001604463, *Planococcus kraunhiae*: LC055463, *Samia ricini*: ABE98256, *Schistocerca gregaria*: ADV17350, *Tribolium castaneum*: EFA02917. Regarding analysis methods, see above.
(JPG)

S3 Fig Structures and expression of Pktai isoforms. A. Protein structure of PkMet with bHLH, PAS and PAS B domains. B. Structure of PkTai with the identified isoforms. Grey: Open Reading Frame. C. Expression profiles of *Pktai* IN-1 and *Pktai* DEL-1. D. Expression profiles of *Pktai* 5A and *Pktai* 5B.
(JPG)

S4 Fig Structure of PkKr-h1. A. Alignment of the zinc-finger region of Krüppel homolog 1 of different insect species. GenBank accession numbers: Bg (*Blattella germanica*): CCC55948, Bm (*Bombyx mori)*: NP_001171332, Dm (*Drosophila melanogaster*): NP_477466, Fo (*Frankliniella occidentalis*): BAJ41257, Pa (*Pyrrhocoris apterus*): AEW22979, Pk (*Planococcus kraunhiae*): LC075597 and LC075598, Tc (*Tribolium castaneum*): NP_001129235. B. Structure of *PkKr-h1* cDNA. Grey: ORF. Scale bar: 200 bp. C. Amino acid alignment of the indel regions for *PkKr-h1* A and *PkKr-h1* B.
(JPG)

S5 Fig Expression profiles of *PkKr-h1* isoforms during male and female development after oviposition. A. *PkKr-h1* A, B. *PkKr-h1* B. E: Egg, N1: 436 first-instar nymph, N2: second-instar nymphs, N3: female third-instar nymph, Pre: 437 male prepupa, Pu: male pupa, m: male adult, f: female adult. Samples were collected 438 from E to N2D3 using the sex-ratio bias strategy (see the Methods section)
(JPG)

S6 Fig Structure of *Pkbr*. A. Alignment of the BTB domain of Br in different insect species. GenBank accession numbers: Bg (*Blattella germanica*): CBJ05857, Of (*Oncopeltus fasciatus*): ABA02191, Tc (*Tribolium castaneum*): NP_001104734, Fo (*Frankliniella occidentalis*): BAJ41241, Dm (*Drosophila melanogaster*): NP_726752, Ad (*Acheta domesticus*): ABA02190, Bm (*Bombyx mori*): NP_001036976, Ap (*Acyrthosiphon pisum*): XP_008180579, *Pkbr1*: LC055465-LC055472, *Pkbr2*: LC055473-LC055475, *Pkbr3*: LC055476-LC055479. B. Structure of *Pkbr1*, C. Structure of *Pkbr2*, D. Structure of *Pkbr3*. Grey: ORF. Scale bar: 100 bp. NZ (non-zinc-finger) means an isoform lacking zinc-finger motifs. Arrows: primers designed for quantitative RT-PCR.
(JPG)

S7 Fig Expression profiles of Pkbr isoforms identified for each of the three *Pkbr* copies. A. *Pkbr1* Z2. B. *Pkbr1* Z4. C. *Pkbr2* Z2. D. *Pkbr2* Z4. E. *Pkbr3* Z2.
(JPG)

S8 Fig Effects of the pyriproxyfen treatment on *Pkbr2*. A. *Pkbr2* common. B. *Pkbr2* Z2. C. *Pkbr2* Z4. PreD1: Treatment 24-48 hours after the prepupal molt, RNA extraction 5 days after the treatment, N = 6. PuD0: Treatment 0-24 hours after the pupal molt, RNA extraction 6 days after the treatment, N = 5. Boxplots constructed with the ggplot2 R package (with upper and lower hinges: 1st and 3rd quartiles, middle line: median, dots: outlier values).
(JPG)

S1 Table List of primers designed for this study. (PDF)

S2 Table Output of Student’s t-test (2-sample and unpaired) using R. (PDF)
