# Supplementary material for: Differential Juvenile Hormone Variations in Scale Insect Extreme Sexual Dimorphism
Source: PLoS One. 2016 Feb 19;11(2):e0149459. doi: 10.1371/journal.pone.0149459 (PMC4760703; doi:10.1371/journal.pone.0149459)
Supplement: S1 Text — (PDF) [file pone.0149459.s001.pdf]

**S1 Text**

Differential juvenile hormone variations in scale insect extreme sexual dimorphism

Isabelle M. Vea[1], Sayumi Tanaka[1], Takahiro Shiotsuki[2], Akiya Jouraku[2], Toshiharu Tanaka[1], Chieka Minakuchi[1]
[1] Graduate School of Bioagricultural Sciences, Nagoya University, Nagoya, Japan
[2] National Institute of Agrobiological Sciences, Tsukuba, Japan

**A) RNA-seq analysis**
Japanese mealybug *P. kraunhiae* culture for RNA-seq analysis was kindly provided by Dr. Mayumi Teshiba at Fukuoka Agriculture and Forestry Research Center. Total RNAs were extracted using ISOGEN from the whole body of nymphs and purified using the SV Total RNA Isolation System. Preparation of cDNA libraries from the total RNAs and sequencing by Illumina HiSeq 2000 sequencer were performed by Macrogen Japan Co., Ltd. (Tokyo, Japan) to give 3867 Mbp of transcriptome data (accession number DRA004114; Sugahara et al., in preparation). The RNA-seq paired-end reads (101 bp per read) were filtered by Trimmomatic to remove adapter sequences and low quality reads. The remaining paired-end reads were de novo assembled by Trinity and 50673 contigs were generated. The GC content, total size, average length, and N50 of the RNA-seq contigs were 38.05%, 72.3 Mbp, 1428 bp, and 2703 bp, respectively.

**B) PCR protocols**

1. RT-PCR: PCR products were obtained from 30 µL of a PCR mixture (0.18 µL ExTaq HS, 3 µL ExTaq buffer, 0.6 µM of each primer, 2.4 µL of 2.5 mM dNTP, and 3 µL cDNA), and the PCR conditions used were as follows: 94ºC 1 min, 36 cycles of (94ºC 1 min, 60ºC 1 min, and 72ºC 1 min), and 72ºC 1 min.
2. RACE PCR: A 5-µL reaction volume containing 0.5 µL of 10x Adv.2 PCR buffer, 0.2 µL of 2.5 mM dNTP, 0.1 µL of 50x Adv. 2 Polymerase Mix, 0.5 µL of 10x universal primer mix, 0.5 µM of gene-specific primers, and 0.2 µL of a cDNA template. The PCR conditions used were 5 cycles of (94ºC 15 sec and 72ºC 3 min), 5 cycles of (94ºC 15 sec, 70ºC 15 sec, and 72ºC 3 min), and 30 cycles of (94ºC 15 sec, 68ºC 15 sec, and 72ºC 3 min).
3. Quantitative RT-PCR: A 14-µL reaction volume containing 7 µL of SYBR Premix Ex Taq (Takara Bio), 0.2 µM of each primer, and 0.5 µL of a cDNA template or plasmid standard. PCR conditions were 95º C for 30 sec, followed by 40 cycles at 95ºC for 5 sec and 60º C for 30 sec. After thermal cycling, the absence of unwanted byproducts was confirmed by a melting curve analysis.

**C) Phylogenetic analyses of protein sequences**
The protein sequences of Broad zinc-finger motifs and the JHAMT conserved region for other insects were obtained from GenBank (accession numbers in figure captions) and aligned using Geneious version 5.1.7 (www.geneious.com, Kearse et al., 2012) with *P. kraunhiae* new sequences. Phylogenetic analyses were then performed using MrBayes (3.2.5) (Ronquist and Huelsenbeck, 2003) for 4 runs of 1 million generations with trees sampled every 1000 generations, using the amino acid mixed model (command line: aamodelpr=mixed). We considered convergence when the average standard deviation split frequency was < 0.05. Trees were unrooted and compiled using the compatibility summary. The Nexus files of Broad and JHAMT datasets with MrBayes command lines are available as separate supplementary files.

**D) Graphs and statistical analyses**

All graphs were obtained using R (version 3.1.2) (R core team, 2015). The significance of gene expression differences (mean) was assessed using the unpaired two-sample Student's t-test. The R script of all analyses pertaining to expression profile graphs and statistical tests are in the first author's GitHub repository (DOI: 10.5281/zenodo.34238).

**References**

Kearse, M., Moir, R., Wilson, A., Stones-Havas, S., Cheung, M., Sturrock, S., Buxton, S., Cooper, A., Markowitz, S., Duran, C., Thierer, T., Ashton, B., Mentjies, P., & Drummond, A. 2012. Geneious Basic: an integrated and extendable desktop software platform for the organization and analysis of sequence data. *Bioinformatics*, 28(12), 1647-1649.

Team, R. Core. 2015 "R: A language and environment for statistical computing. Vienna, Austria; 2014." URL http://www. R-project. org (2015).

Ronquist, F. and J. P. Huelsenbeck. 2003. MRBAYES 3: Bayesian phylogenetic inference under mixed models. *Bioinformatics* 19:1572-1574.
