# Supplementary figures and images for: Differential Juvenile Hormone Variations in Scale Insect Extreme Sexual Dimorphism
Source: PLoS One. 2016 Feb 19;11(2):e0149459. doi: 10.1371/journal.pone.0149459 (PMC4760703; doi:10.1371/journal.pone.0149459)

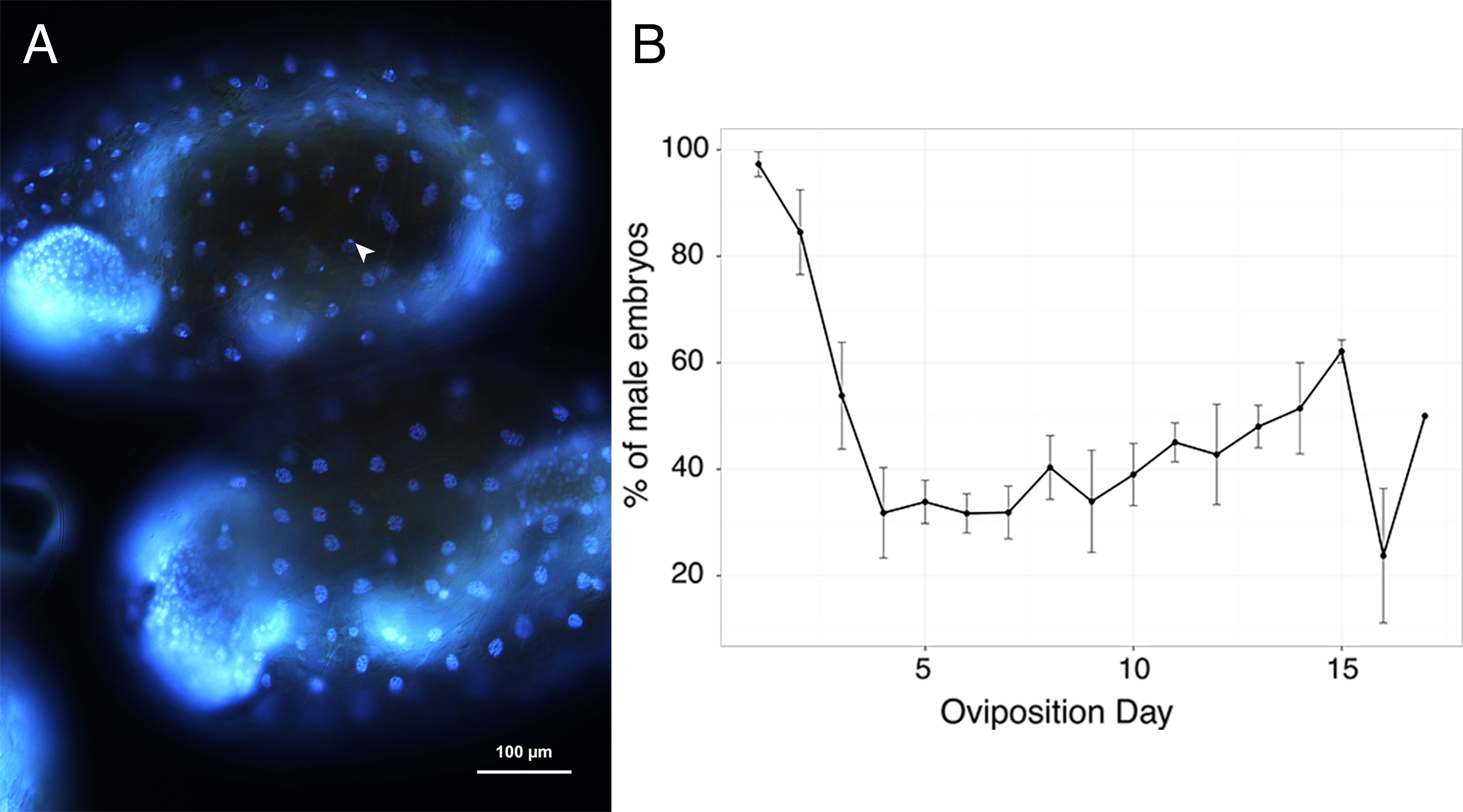

Supplement: S1 Fig — A. Photograph of fixed and DAPI-stained embryos at oviposition, top: male embryo as evidenced by the bright, condensed paternal genome, bottom: female embryo as evidenced by homogeneous nuclei. B. Percentage of male embryos on each oviposition day. Counts were made using DAPI-stained embryos collected every 24 hours from fertilized females (N = 5) and observed an Olympus BX41 Fluorescence microscope (x200). (JPG) [file pone.0149459.s002.jpg]

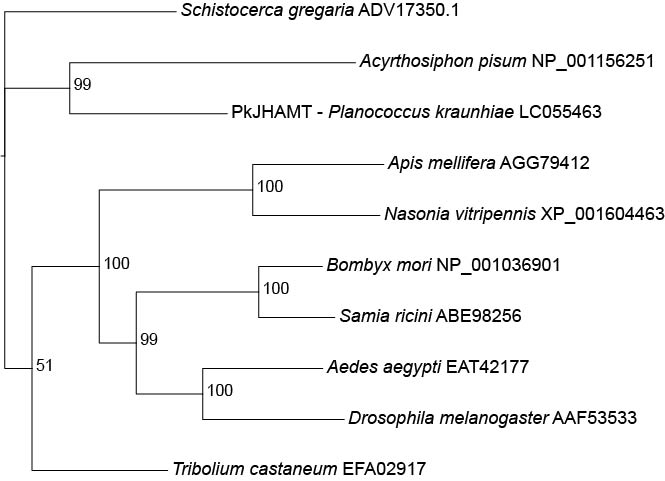

Supplement: S2 Fig — GenBank accession numbers: Acyrthosiphon pisum: NP_001156251, Aedes aegypti: EAT42177, Apis mellifera: AGG79412, Bombyx mori: NP_001036901, Drosophila melanogaster: AAF53533, Nasonia vitripennis: XP_001604463, Planococcus kraunhiae: LC055463, Samia ricini: ABE98256, Schistocerca gregaria: ADV17350, Tribolium castaneum: EFA02917. Regarding analysis methods, see above. (JPG) [file pone.0149459.s003.jpg]

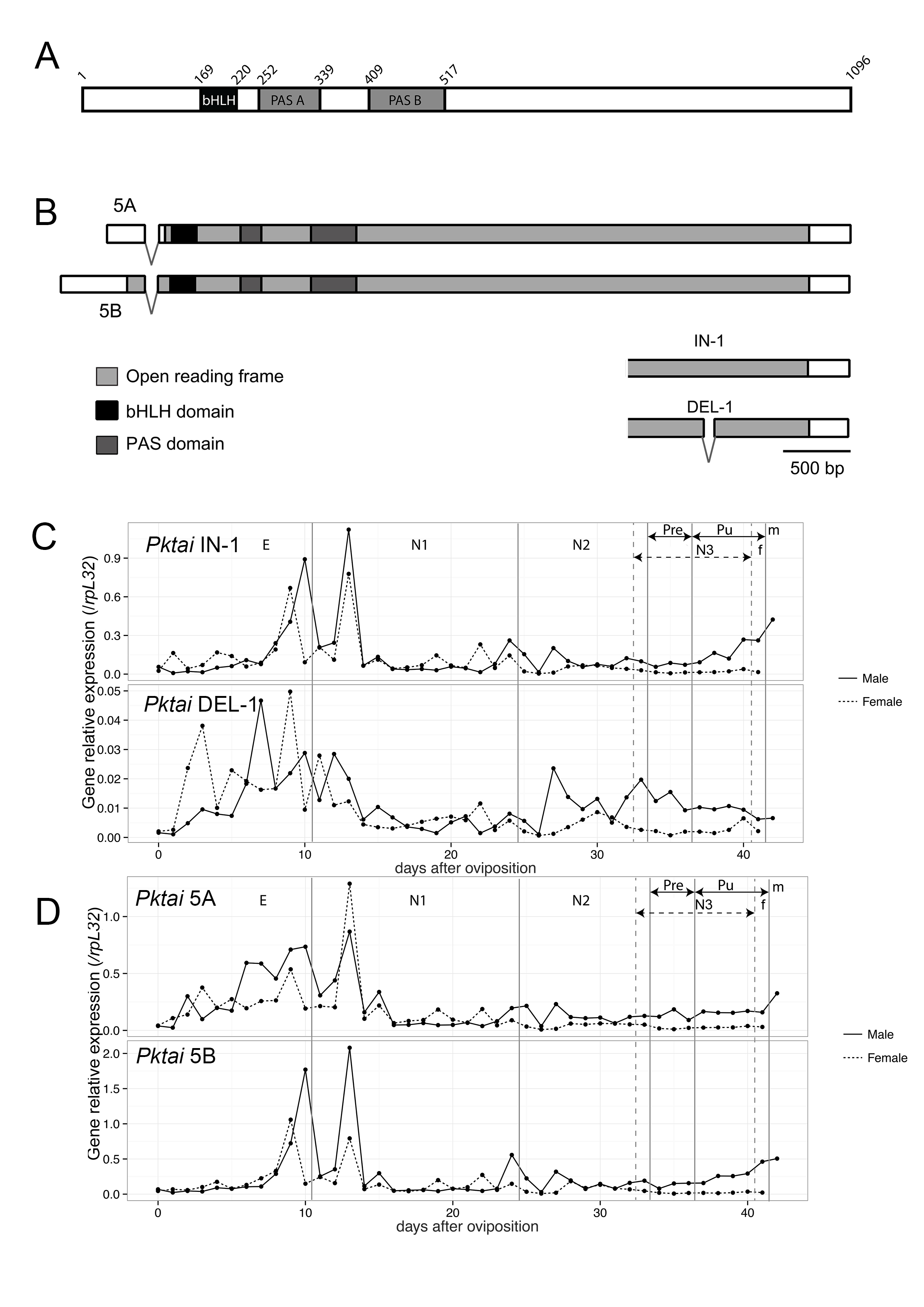

Supplement: S3 Fig — A. Protein structure of PkMet with bHLH, PAS and PAS B domains. B. Structure of PkTai with the identified isoforms. Grey: Open Reading Frame. C. Expression profiles of Pktai IN-1 and Pktai DEL-1. D. Expression profiles of Pktai 5A and Pktai 5B. (JPG) [file pone.0149459.s004.jpg]

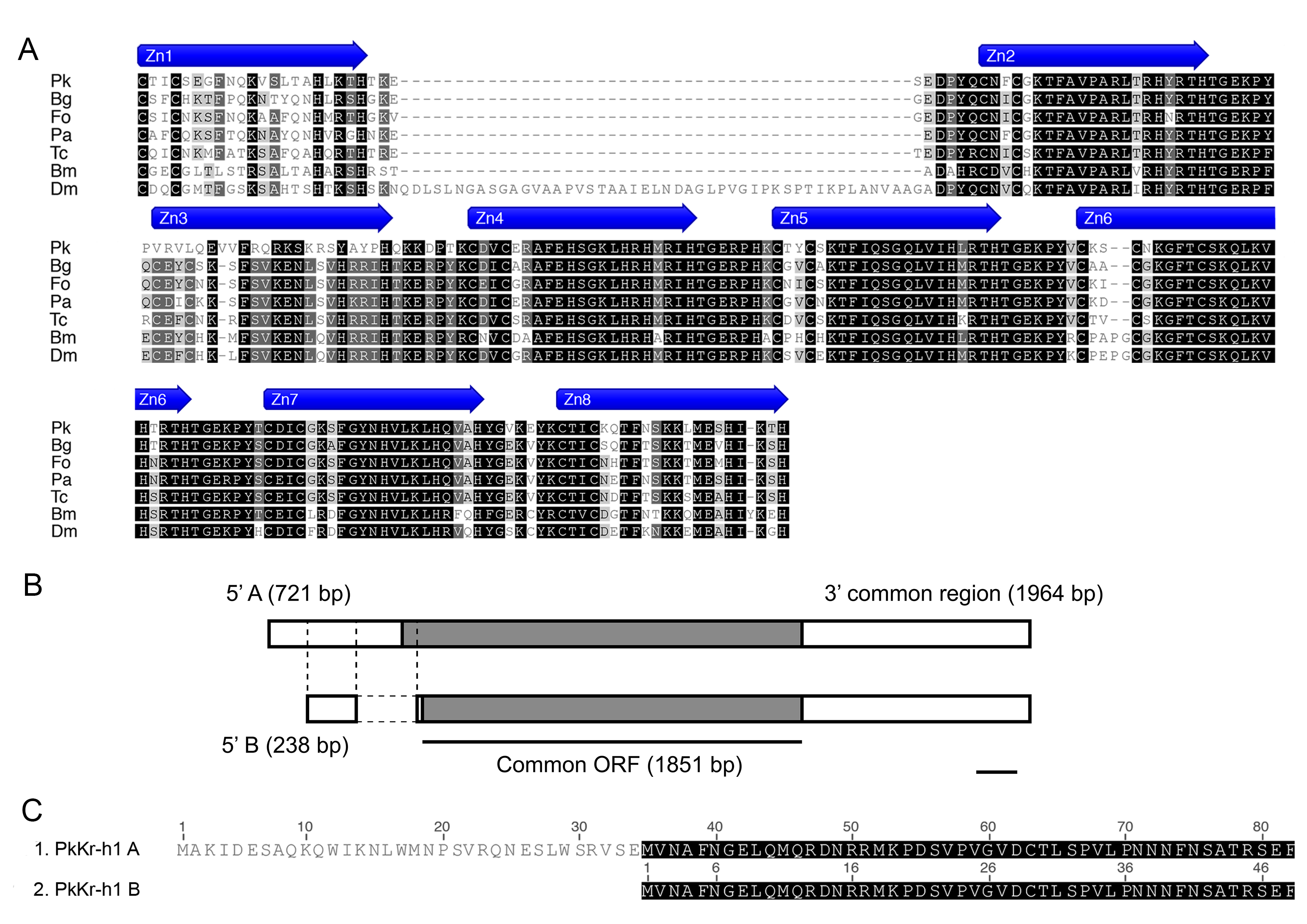

Supplement: S4 Fig — A. Alignment of the zinc-finger region of Krüppel homolog 1 of different insect species. GenBank accession numbers: Bg (Blattella germanica): CCC55948, Bm (Bombyx mori): NP_001171332, Dm (Drosophila melanogaster): NP_477466, Fo (Frankliniella occidentalis): BAJ41257, Pa (Pyrrhocoris apterus): AEW22979, Pk (Planococcus kraunhiae): LC075597 and LC075598, Tc (Tribolium castaneum): NP_001129235. B. Structure of PkKr-h1 cDNA. Grey: ORF. Scale bar: 200 bp. C. Amino acid alignment of the indel regions for PkKr-h1 A and PkKr-h1 B. (JPG) [file pone.0149459.s005.jpg]

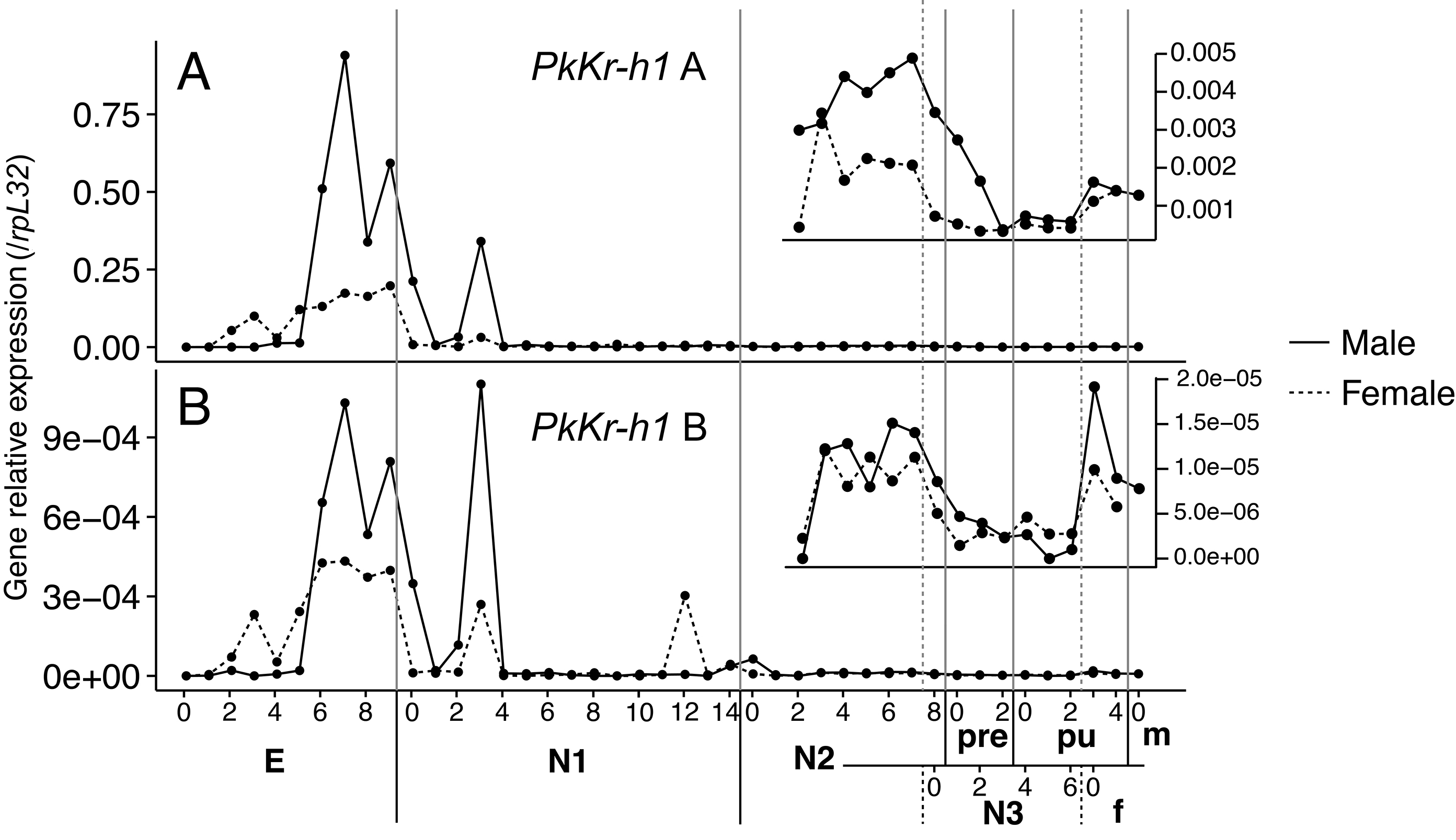

Supplement: S5 Fig — A. PkKr-h1 A, B. PkKr-h1 B. E: Egg, N1: 436 first-instar nymph, N2: second-instar nymphs, N3: female third-instar nymph, Pre: 437 male prepupa, Pu: male pupa, m: male adult, f: female adult. Samples were collected 438 from E to N2D3 using the sex-ratio bias strategy (see the Methods section) (JPG) [file pone.0149459.s006.jpg]

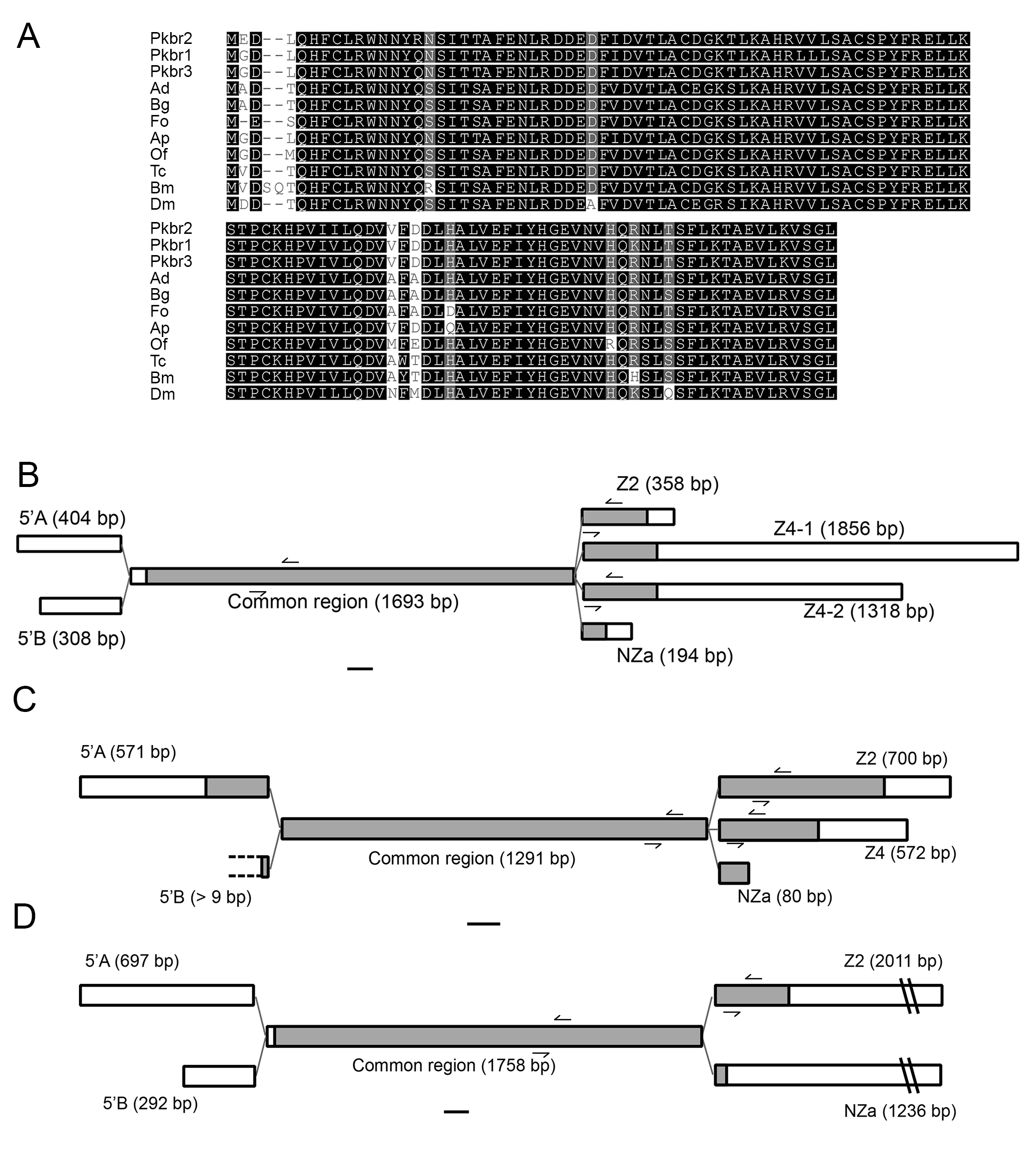

Supplement: S6 Fig — A. Alignment of the BTB domain of Br in different insect species. GenBank accession numbers: Bg (Blattella germanica): CBJ05857, Of (Oncopeltus fasciatus): ABA02191, Tc (Tribolium castaneum): NP_001104734, Fo (Frankliniella occidentalis): BAJ41241, Dm (Drosophila melanogaster): NP_726752, Ad (Acheta domesticus): ABA02190, Bm (Bombyx mori): NP_001036976, Ap (Acyrthosiphon pisum): XP_008180579, Pkbr1: LC055465-LC055472, Pkbr2: LC055473-LC055475, Pkbr3: LC055476-LC055479. B. Structure of Pkbr1, C. Structure of Pkbr2, D. Structure of Pkbr3. Grey: ORF. Scale bar: 100 bp. NZ (non-zinc-finger) means an isoform lacking zinc-finger motifs. Arrows: primers designed for quantitative RT-PCR. (JPG) [file pone.0149459.s007.jpg]

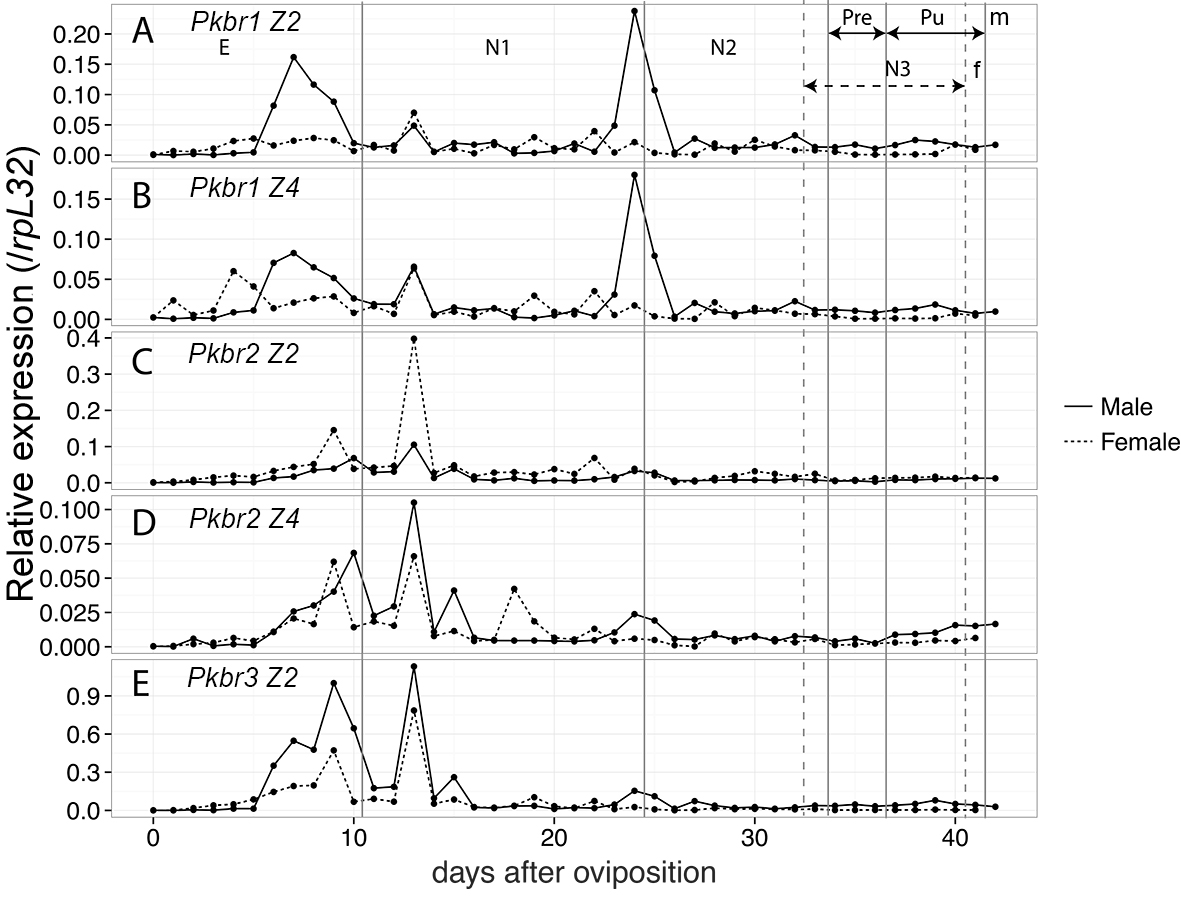

Supplement: S7 Fig — A. Pkbr1 Z2. B. Pkbr1 Z4. C. Pkbr2 Z2. D. Pkbr2 Z4. E. Pkbr3 Z2. (JPG) [file pone.0149459.s008.jpg]

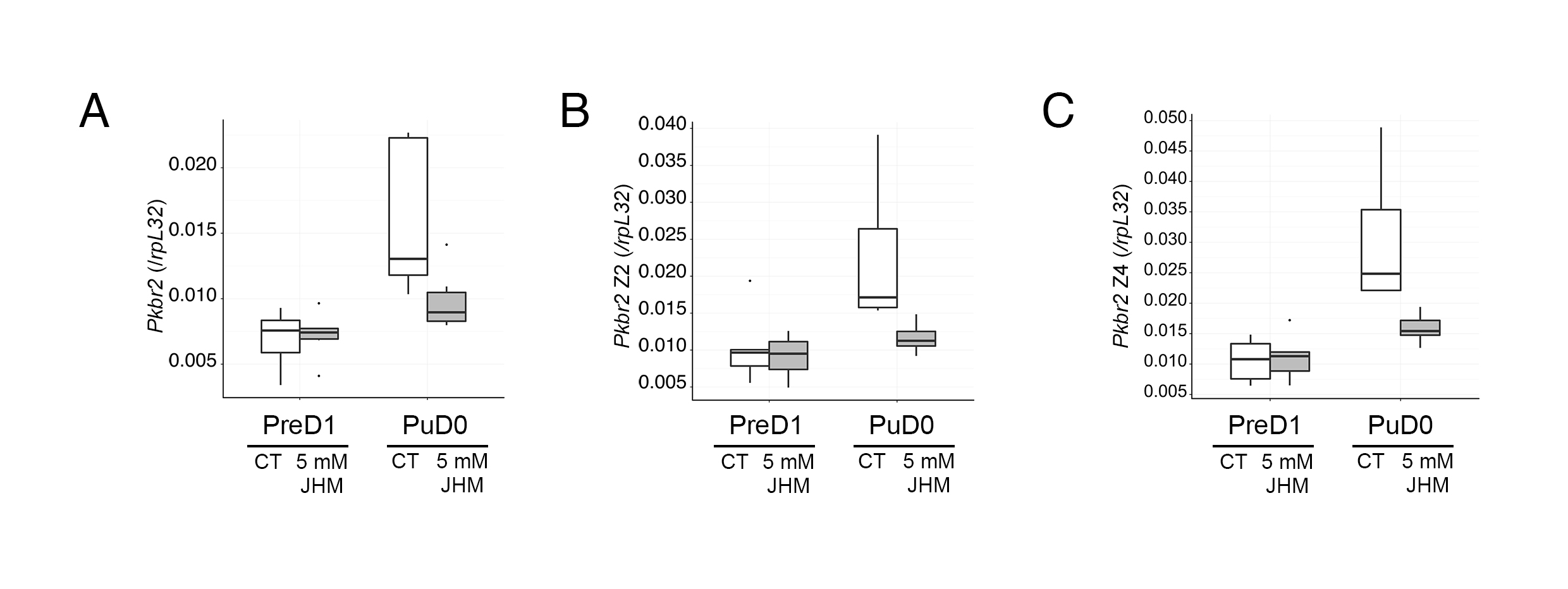

Supplement: S8 Fig — A. Pkbr2 common. B. Pkbr2 Z2. C. Pkbr2 Z4. PreD1: Treatment 24-48 hours after the prepupal molt, RNA extraction 5 days after the treatment, N = 6. PuD0: Treatment 0-24 hours after the pupal molt, RNA extraction 6 days after the treatment, N = 5. Boxplots constructed with the ggplot2 R package (with upper and lower hinges: 1st and 3rd quartiles, middle line: median, dots: outlier values). (JPG) [file pone.0149459.s009.jpg]
