# Supplementary material for: Differential Juvenile Hormone Variations in Scale Insect Extreme Sexual Dimorphism
Source: PLoS One. 2016 Feb 19;11(2):e0149459. doi: 10.1371/journal.pone.0149459 (PMC4760703; doi:10.1371/journal.pone.0149459)
Supplement: S1 Table — (PDF) [file pone.0149459.s010.pdf]

S1 Table List of primers used in the present study

Region	Primer name	Sequence 5'->3'	Used for	Fragment size bp
rpL32	Pkrp49_ORF-F1	ATGTCTATTAAACCGAAGTACC	Cloning	405
	Pkrp49_ORF-R1	TTACTCTGCCTCTTCACTTCGTA		--
	Pkrp49-QF2	CCATCAAAGTGACCGCTATGTC	qRT-PCR	127
	Pkrp49-QR2	CGCGTTACTTCCATATCCAACA		--
	Pkrp49-RR1	TGGCCCTTGAACCTTCTTCGTAC TCTGT	5' RACE	
	Pkrp49-RF1	CAGAGTACGAAGAAGGTTCAAG GGCCAGT	3' RACE	
Methoprene-tolerant	PkMet-F1	TATTTCACGGATTGCGCTTACG	Cloning	3346
	PkMet-R1	CAGCCAGCCTCGAATAAGAGC		--
	PkMet-QF1	TAATACCAACGCCAACAACTGC	qRT-PCR	122
	PkMet-QR1	CGTTTAAATGCATCGGTTCGTA		--
	PkMet-RR1	TCCACCCCATTTCGTAAGCGCAA TC	5' RACE	
	PkMet-RF1	CAACGCATCAGAATGTCGGCGT GTAG	3' RACE	
taiman	PkTai-F1	TAATGAAAAACGTCGCCGAGAA	Cloning	2674
	PkTai-R1	ACTGTTGCTGTTGCATTTGCTG		--
	PkTai-RR1	TGACGGAGATTAACTCGGCCAG CTCT	5' RACE	
	PkTai-RF1	TTCCTCAACGAGCTGTTGCAGTC GAG	3' RACE	
	PkTai-ID-F1	ATATCTCGCCCACCACTGCTAC	Cloning	795 and 594
	PkTai-ID-R1	AGAGCGAGAGTGAAAACGAAGG		
	PkTai-QF1	CCGCAACAAACAGATACTGGAG	qRT-PCR	121
	PkTai-QR1	GTTCATTGAAGACGGTGTGGTC		--
	PkTai-IN1-QF1	CTGCATACGAGCGTATTCTTCG	qRT-PCR	122
	PkTai-IN1-QR1	AAGATTCGCCGTGATGTTGATA		--
	PkTai-DEL1-QF2	CTTCGCAAGTACCACCAGGC	qRT-PCR	109
	PkTai-DEL1-QR1	TGTCTGGCAGTTACCACAGTCC		--
	PkTai_5A-QF1	CGTGGTATTTCGCGTATGATTG	qRT-PCR	125
	PkTai_5A-QR1	GTAACCACTGCACGGCATTTT		--
	PkTai_5B-QF1	CTCTTGATGGTTGGAATTCGTC	qRT-PCR	114
	PkTai_5B-QR1	TTGCTAGACGATTTTCGCATTG		--
jhamt	PkJHAMT-F1	ATGACTTCAGGTGCACTATTCAG AC	Cloning	840
	PkJHAMT-R1	TTAGTGTGGTTTATAGGCGTAAA TCG		
	PkJHAMT-QF1	TCATGGAATGCAGGACAGAGAT	qRT-PCR	104
	PkJHAMT-QR1	CAGGACCGCAACCAATATCTAA		
	PkJHAMT-RR1	GCGATGTCAGCATCTCTGTCCTG	5' RACE	

		CAT		
	PkJHAMT-RF1	CGACCAAGATTGGAAGGAGAAG ATGTGG	3’ RACE	
Krüppel homolog 1	PkKr-h1-F1	ATGGCAAAAATTGATGAGAGTG CT	Cloning	1956
	PkKr-h1-R1	TTACGACGAAGCTTTCGCGTACT G		
Krüppel homolog1, common region	Pk-kr-h1-F	GCTTTTCGCCGGTATGTGTT	qRT-PCR	149
	Pk-kr-h1-R	CGAACGAGCCTTCGAACATT		
	PkKr-h1-RR1	TGCGTTTTTAGATGCGCCGTCAG ACT	5’ RACE	
	PkKr-h1-RR2	CGACTCCAACAGGTACAGAATCT GGCTTCA	5’ RACE	
	PkKr-h1-RF1	TGCTGAAGTTGCATCAGGTGGCT CAT	3’ RACE	
	PkKr-h1-RF2	CGTCGATCTCGCAGTCAACAAAG ACG	3’ RACE	
PkKr-h1 A and B	PkKrh1-5A_QF1	GAGAGTGCTCAAAAACAGTGGA TT	qRT-PCR	125
	PkKrh1-5AB_QR1	CTTTGCATTTGCAGTTCTCCAT		
	PkKrh1-5B_QF1	GACGTGTTCATATTATACCAAT	qRT-PCR	83 (with PkKrh1-5AB_QR1 primer)
BR COPIES				
Pkbr1 com	Pkbr7969-QF1	AATTATCCCAGAACATCATTAA	qRT-PCR	132
	Pkbr7969-QR1	AGCTGATTTGGCTCGTTTCAA		--
Pkbr2 com	Pkbr8968-QF1	TGAGTGATCAGTCGTCACCTGT	qRT-PCR	110
	Pkbr8968-QR1	CATGTTGAATCTCAAAGACGCC		--
Pkbr3 com	Pkbr19426-QF1	CTGCCTCTAGCATCAGCATCAC	qRT-PCR	112
	Pkbr19426-QR1	GTTGCAGATGTTGAGCATTGTG		--
ISOFORMS				
*Pkbr1*_Z2	Pkbr7969_3a-QF1	GTCTGGCTTGTGGCAAAGTGT	qRT-PCR	117
	Pkbr7969_3a-QR1	AGCAGTAAACACGCTCGCAAAT A		--
*Pkbr1*_Z4	Pkbr7969_3b-QF1	AGTGGCAATCAGGGAAAAGGAG	qRT-PCR	127
	Pkbr7969_3b-QR1	GTTGCTCAATATGACGTTTCAAG		--
*Pkbr2*_Z2	Pkbr8968_3a-QF1	ATGTCTGGCTTGTGGCAAAGTAC	qRT-PCR	118
	Pkbr8968_3a-QR1	GCAGTAAACGCGCTCACAAATC		--
*Pkbr2*_Z4	Pkbr8968_3b-QF2	GTCGCAATCAGGGAAAAGGAA	qRT-PCR	123
	Pkbr8968_3b-QR2	TCAATGTCAGTCGAGTGCTCAGA		--
*Pkbr3*_Z2	Pkbr19426_3a-QF1	ATGTCTGGCTTGTGGTAAAGTT	qRT-PCR	119
	Pkbr19426_3a-QR1	AGCAATAAACACGCTCGCAAAT T		--
Pkbr1	Pkbr-7969-RR1	CAGTTTTCAAAAACGATGTCAAA TTCT	5’ RACE	

	Pkbr-7969-RF2	ATGACCACTTCTACAGCTGCCAC TACC	3’ RACE	
	Pkbr14866-RF1	TGGCAATCAGGGAAAAGGAGTA GGAGAAGG	3’ RACE	
Pkbr2	Pkbr-8968-RR1	CTGTTTTCAAAAACGAGGTCAAG TTTC	5’ RACE	
	Pkbr8968-2,3-RR1	TGATCACTGGATGCTTACAGGGT GTACTGA	5’ RACE	
	Pkbr-8968-RF1	CAACAATATGGTAATTTCAACCG TCG	3’ RACE	
Pkbr3	Pkbr19426-RR1	GCGACTTTGTTGAGAAGCGTGCT AATAG	5’ RACE	
	Pkbr21412-RR1	GCGTTTGAGCATTCCAGTACTAC CACGATG	5’ RACE	
	Pkbr21412-RR2	CCACGATGCTGCAGGCTTTTATG AGTTG	5’ RACE	
	Pkbr19426-RF1	CAAAATGCAGCAGCCACCACTA CAACA	3’ RACE	
	Pkbr19426-RF4	CTAGCAGTGGGGTTGGTGGCAG CTC	3’ RACE	
