# Supplementary material for: Differential Juvenile Hormone Variations in Scale Insect Extreme Sexual Dimorphism
Source: PLoS One. 2016 Feb 19;11(2):e0149459. doi: 10.1371/journal.pone.0149459 (PMC4760703; doi:10.1371/journal.pone.0149459)
Supplement: S2 Table — (PDF) [file pone.0149459.s011.pdf]

S2 Table Output of the Student's *t*-test (2-sample and unpaired)

Stage	Gene	t	df	p-value	95% confidence interval	Control		Treatment	
						Mean	SD	Mean	SD
Prepupa	*PkMet*	-1.6773	6.511	0.1406	-0.11309954; 0.02007199	0.1384557	0.02486491	0.1849695	0.06321316
Pupa		-0.4667	7.991	0.6532	-0.7066012; 0.4687802	0.5103808	0.29488047	0.6292913	0.53404007
Prepupa	*Pktai*	-2.1554	8.739	0.0604	-0.243691140; 0.006444042	0.1983923	0.07506666	0.3170158	0.11197459
Pupa		1.3399	5.364	0.2342	-0.5779953; 1.8915831	1.1273003	1.01266765	0.4705064	0.45949615
Prepupa	*Pktai* IN-1	-2.724	6.096	0.03391	-0.25596429 -0.01420593	**0.1232682**	**0.03839298**	**0.2583533**	**0.11524342**
Pupa		1.794	6.932	0.1163	-0.1897969; 1.3734942	1.0309839	0.62414504	0.4391353	0.43072152
Prepupa	*Pktai* DEL-1	-4.6291	8.737	0.001339	-0.023977244; -0.008186897	**0.01284740**	**0.004737565**	**0.02892948**	**0.007069149**
Pupa		0.536	8.797	0.6053	-0.02430555 0.03932915	0.02676884	0.022597383	0.01925704	0.023787748
Prepupa	*PkKr-h1*	-9.185	5.012	0.0002533	-0.3965005; -0.2231941	**0.00498346**	**0.002879019**	**0.31483074**	**0.082581299**
Pupa		-1.86	5.053	0.1214	-0.8812110; 0.1399882	0.03489796	0.032228752	0.40550937	0.486791563
Prepupa	*PkKr-h1* A	-7.3395	5.005	0.0007335	-0.12333790; -0.05936676	**0.001215437**	**0.0006762445**	**0.092567766**	**0.0304803486**
Pupa		-1.7897	5.111	0.1322	-0.08344160; 0.01467836	0.005666724	0.0045132693	0.040048344	0.0467954001
Prepupa	*PkKr-h1* B	0.7175	5.056	0.5048	-0.001185953; 0.002108668	0.0006530517	0.0015705504	0.0001916943	0.0001174338
Pupa		0.606	5.605	0.5682	-0.001198421 0.001969711	0.0007613997	0.0012972453	0.0003757547	0.0006404445
Prepupa	*Pkbr1*	1.1072	5.026	0.3184	-0.06710946 0.16893629	0.058924830	0.112492088	0.008011414	0.005737789
Pupa		1.3187	5.005	0.2444	-0.007291479 0.022661252	0.010463914	0.012282630	0.002779028	0.004768020
Prepupa	*Pkbr1* Z2	0.01017740	8.339	0.7768	-0.02372600 0.01834553	0.02514022	0.01184079	0.02783046	0.01913589
Pupa		3.6973	7.826	0.006305	0.00953160 0.04145515	**0.03826052**	**0.01184079**	**0.01276715**	**0.01017740**
Prepupa	*Pkbr1* Z4	-0.4274	9.418	0.6787	-0.02050683 0.01395205	0.02482666	0.01151242	0.02810405	0.01484070
Pupa		2.3667	8.339	0.04425	0.0006945604 0.0420761484	**0.04191781**	**0.01114936**	**0.02053245**	**0.01845886**
Prepupa	*Pkbr2*	-1.5357	9.231	0.1581	-0.007359867 0.001394197	0.007522514	0.002837463	0.010505349	0.003818841
Pupa		-0.3209	6.731	0.758	-0.02417918 0.01844208	0.019745933	0.007894325	0.022614479	0.020117051
Prepupa	*Pkbr2* Z2	-0.4904	9.605	0.6348	-0.010605108 0.006796351	0.01152830	0.007376588	0.01343268	0.006004955
Pupa		0.0368	8.151	0.9715	-0.03268955 0.03375405	0.02926386	0.017225892	0.02873161	0.029954805
Prepupa	*Pkbr2* Z4	-1.4389	9.861	0.1812	-0.010683953	0.01152882	0.0047315	0.0157162	0.00533153

					0.002309192		31	0	3
Pupa		0.1123	8.052	0.9133	-0.03795135 0.04184242	0.03932104	0.0202715 06	0.0373755 1	0.03615153 6
Prepupa	*Pkbr3*	-3.8123	6.003	0.008832	-0.3289491 - 0.0717742	**0.1356336**	**0.0390434 6**	**0.3359952**	**0.12267347**
Pupa		-1.1011	6.041	0.3128	-0.5622983 0.2128865	0.2097946	0.1102849 2	0.3845005	0.36937731
Prepupa	*Pkbr3* Z2	-3.9576	6.487	0.006386	-0.15814943 - 0.03865075	**0.07161787**	**0.0221320 5**	**0.1700179 6**	**0.05674008**
Pupa		-1.3502	5.672	0.2283	-0.28227621 0.08334711	0.09957450	0.0416099 6	0.1990390 5	0.17459151
